# Sodium alginate (SA)-based adsorbents for the adsorption of water pollutants: recent advancements, mechanisms, challenges, and future directions

**DOI:** 10.1039/d6ra04069a

**Published:** 2026-07-21

**Authors:** Yawen Li, Humaira Parveen, Sayeed Mukhtar, Mona O. Albalawi, Ibrahim Saleem Alatawi, Uzma Faridi, Yan Xu, Sajjad Hussain, Irfan Ijaz

**Affiliations:** a School of Pharmacy, Xinxiang University Xinxiang 453003 Henan P. R. China; b School of Pharmacy, Key Laboratory of Nano-carbon Modified Film Technology Engineering, Henan Province, Xinxiang University Xinxiang 453003 Henan P. R. China; c Department of Chemistry, Faculty of Science, University of Tabuk Tabuk 71491 Saudi Arabia; d Department of Biochemistry, Faculty of Science, University of Tabuk Tabuk 71491 Saudi Arabia; e School of Chemistry, Faculty of Basic Sciences and Mathematics, Minhaj University Lahore Lahore 54700 Pakistan

## Abstract

The presence of emerging contaminants in water bodies poses a significant threat to both environmental and human health. Conventional wastewater treatment approaches often face sustainability challenges. Among various approaches, adsorption is recognized as an effective method for removing emerging pollutants, such as heavy metal ions, radioactive metal ions, antibiotics, phosphate ions, dyes, fertilizers, and pesticides, owing to its high selectivity, minimal secondary pollution, scalability, and operational simplicity. This review article comprehensively discusses the recent advancement in the design of sodium alginate (SA)-based adsorbents for wastewater treatment. Sodium alginate (SA), a natural, linear, and unbranched polysaccharide, has emerged as a promising adsorbent for wastewater treatment due to its excellent properties, including abundant functional groups (hydroxyl and carboxylic groups), high hydration, degradability, outstanding biocompatibility, and customizable physicochemical characteristics. The incorporation of SA into carbon-based materials (*e.g.*, biochar, graphene oxide, and carbon nanotubes), MXenes, MOFs, clay minerals, and other biomolecules (*e.g.*, cellulose, polyethyleneimine, and chitosan) significantly enhances adsorption performance. Then, a possible adsorption mechanism is discussed in terms of hydrogen bonding, surface complexation, electrostatic interactions, and ion exchange. Finally, the challenges and future directions for developing a high-performance SA-based adsorbent are outlined. This article aims to provide researchers with an in-depth framework for understanding, facilitate the widespread use of SA-based adsorbents in water pollution management, and offer significant insights and guidance for future research.

## Abstract 2

The presence of emerging contaminants in water bodies poses a significant threat to both environmental and human health.

## Graphical abstract

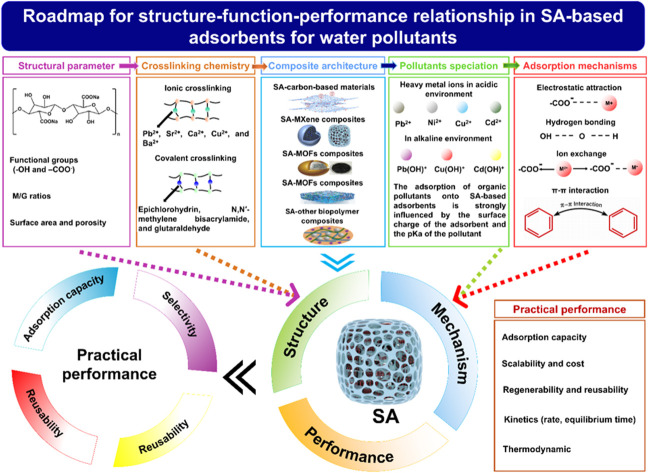

## 1. Introduction

Various chemical substances are categorized as emerging pollutants, including organic dyes, heavy metal ions, antibiotics, and pesticides.^1–3^ Water pollution results from various anthropogenic activities, including unsuitable disposal of drugs, hospital and domestic sewage, agrochemicals, and industrial processes.^4,5^ Water pollution from emerging pollutants is a global concern owing to their low degradability, bioaccumulation, and multiple adverse effects on human health, animals, the microbiota, and aquatic life.^6,7^ Over two billion people lack access to drinkable water due to problems with water quality, and about 3 575 000 people die each year from contagious diseases brought on by contaminated water.^8^ Access to safe drinking water is one of the 17 Sustainable Development Goals (SDGs). By 2050, more than 6 billion people are projected to be affected by a lack of potable water.^9,10^ Thus, adopting eco-friendly, cost-effective, recyclable, and efficient approaches to remove these emerging contaminants is highly desirable. Therefore, numerous approaches, including adsorption, ultrafiltration,^11^ solvent extraction,^12^ ion exchange,^13^ chemical precipitation,^14^ electrodialysis,^15^ reverse osmosis,^16^ and coagulation–flocculation ^17^ have been widely employed for pollutant removal. These strategies generate secondary contaminants, high energy consumption, and high operating and maintenance costs. These challenges limit their practical applications.^18^ Among these approaches, adsorption is widely employed due to no secondary pollution, low cost, simple operation, high efficiency, and other benefits.^19,20^

**Fig. 1:**
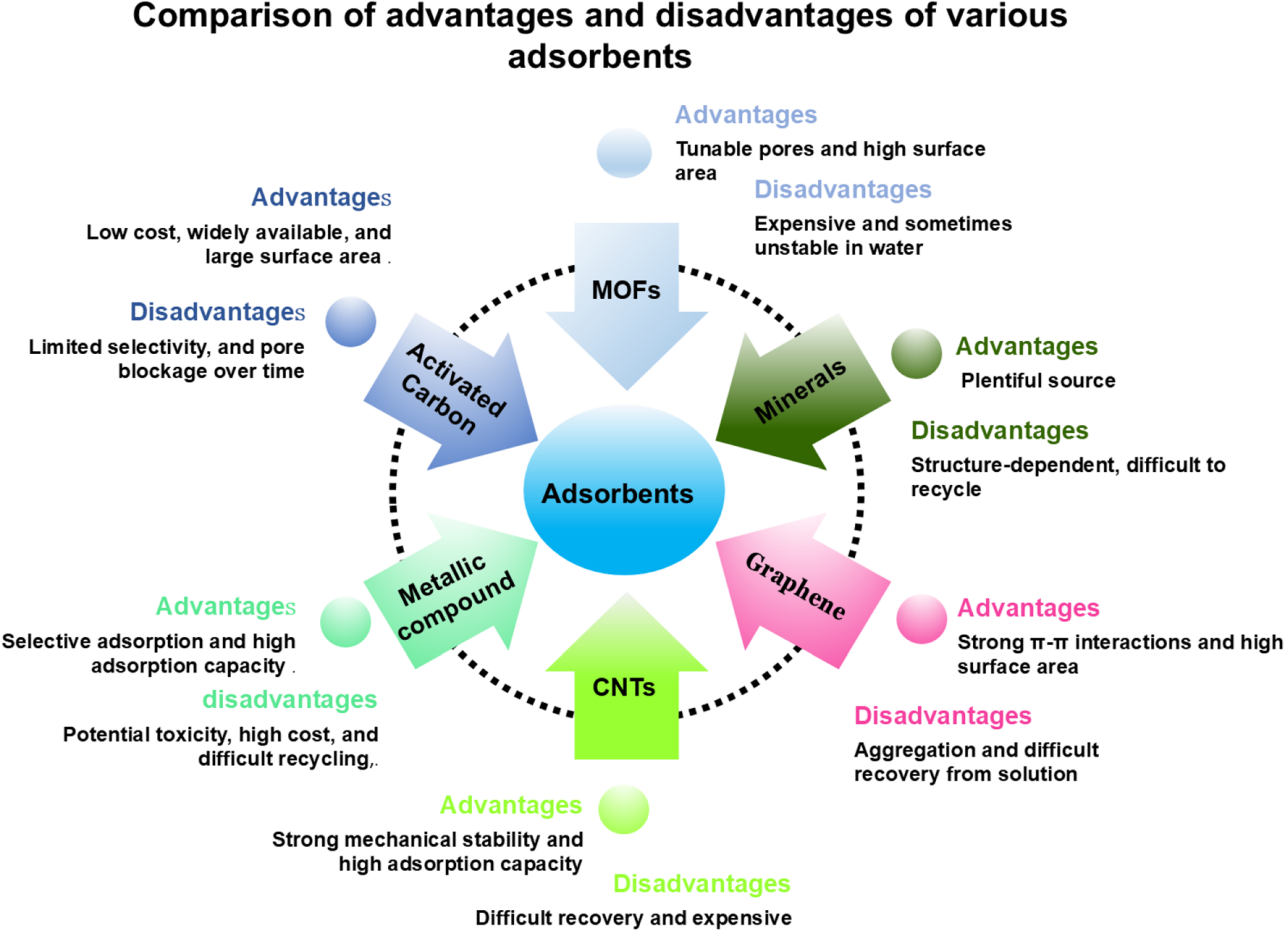
Comparison of the advantages and disadvantages of various adsorbents.

The selection of the adsorbent material is critical for contaminant adsorption, and external adsorption conditions (including interfering ions, temperature, and pH) can also considerably influence adsorption performance.^21,22^ Conventional adsorbents, such as natural minerals,^23,24^ metal–organic frameworks (MOFs),^25^ graphene^26,27^ activated carbon,^28^ metallic compound,^29^ and carbon nanotubes (CNTs)^30,31^ commonly face various challenges, including limited adsorption capacity, difficult recovery, poor selectivity, aggregation, and poor renewability. Fig. 1 illustrates the advantages and disadvantages of various adsorbents. Thus, as research on eco-friendly and sustainable materials advances, alginate-based materials have gained significant attention as a novel adsorbent owing to their ease of functionalization, abundant functional groups, low toxicity, and biodegradability.

SA is a water-soluble, linear anionic, unbranched polysaccharide consisting of β-d-mannuronic acid and (1,4)-linked α-l-guluronic units. Furthermore, many functional groups in its structure, including carboxyl (–COOH) and hydroxyl (–OH) groups, provide more active sites for the removal of emerging contaminants.^32^ Despite these advantages, the heat resistance, stability, and mechanical strength of SA are comparatively low.^33,34^ Thus, chemical and physical modifications are commonly applied to increase its applicability for the adsorption of metal ions, radioactive metal ions, antibiotics, phosphate ions, dyes, phosphate, and pesticides. Traditionally, modification approaches of SA-based adsorbents include cross-linking, surface grafting, and compositing with other materials such as MOFs, MXene, carbonaceous materials, clay minerals, and various biopolymers. The compositing of SA with other substances enhances the physical properties and adsorption capacities of the resulting materials.

**Fig. 2:**
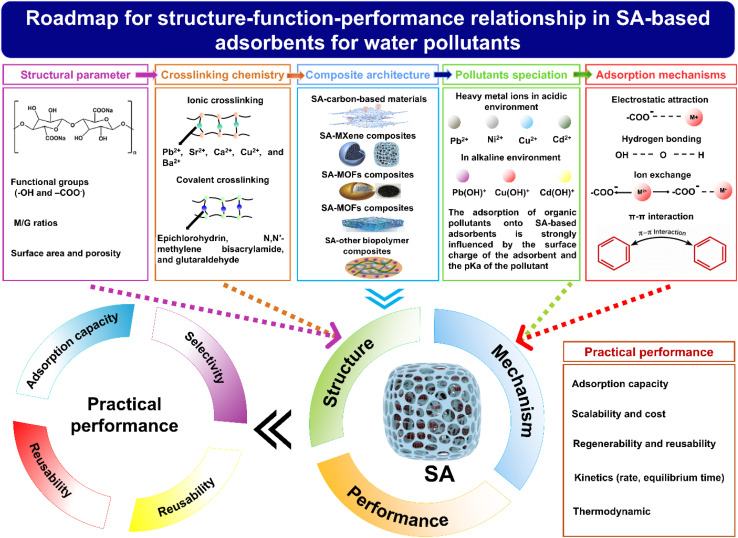
Roadmap for structure–function–performance relationship in SA-based adsorbents for water pollutants.

Despite numerous outstanding reviews that discussed SA-based adsorbents for distinct categories of pollutants, pesticides, heavy metals, and phosphate,^35–38^ or have concentrated on specific material systems such as hydrogels and composites, a comprehensive framework that connects the structural design of sodium alginate adsorbents with their adsorption properties across various pollutant classes is still lacking. Furthermore, recent progress in pollutant speciation, composite architecture, crosslinking, and adsorption mechanisms has not been thoroughly integrated into a single design framework. Such a framework could guide the rational preparation of high-performance sodium alginate adsorbents. To address these gaps, this review offers a thorough overview of the structure, function, and performance of sodium alginate adsorbents used in water treatment. It systematically links: (a) the structural properties of SA, such as molecular structure, functional groups, and M/G ratio; (b) various cross-linking approaches, such as physical or ionic, and chemical/covalent; (c) composite structures, including SA-carbon-based materials (*e.g.*, biochar, graphene oxide, and carbon nanotubes), SA-MXene, SA-MOFs, SA-clay minerals, and other SA-biomolecules (*e.g.*, cellulose, polyethyleneimine, and chitosan); (d) pollutant speciation and physicochemical properties that influence adsorption; (e) adsorption mechanisms, such as ion exchange, surface complexation, electrostatic attraction, hydrogen bonding, π–π interactions, and surface precipitation; and (f) practical performance indicators, such as adsorption capacity, regeneration, and stability, in real-world water treatment, as exhibited in Fig. 2. Finally, challenges and future directions are outlined for developing a high-performance SA-based adsorbent. This article aims to provide researchers with an in-depth knowledge framework, facilitate the extensive utilization of SA-based adsorbents in water pollution management, and offer significant insights and guidance for future research.

## 2. Source and structure of sodium alginate

### 2.1. Source

Industrial SA is primarily obtained from *Phaeophyceae* (brown seaweed) and is predominantly found within the cell walls of brown algae. At the same time, a smaller but essential amount is produced by certain bacteria, including *Pseudomonas* and *Azotobacter*.^39^ Commercially, alginate is prepared from several species of brown seaweeds, including *Sargassum* spp, *Durvillea antarctica*, *Lessonia nigrescens*, *Laminaria japonica*, *Eclonia maxima*, *Ascophyllum nodosum*, *Macrocystis pyrifera*, *Laminaria digitata*, and *Laminaria hyperborea*.^40,41^ However, the composition and yield of alginates depend on multiple parameters, such as environmental conditions. Age of the algae, tissue type, and the species. Seasonal changes and geographic location influence the ratio of mannuronic to guluronic acid units and the amount of extractable alginate, which affect the reaction with metal ions, viscosity, solubility, and gel-forming properties of alginate.^42^ For example, research on *Ecklonia radiata* in New Zealand reported alginate levels ranging from 16.6% to 22.7% of dry weight, with substantial differences across locations and seasons.^43^ Similarly, the M/G ratio of SA for *Sargassum miyabei* decreased from March to August as a result of an increase in the amount of α-l-guluronic acid (G) unit with time. Moreover, alginate from species that typically inhabit cold waters exhibits insufficient viscosity.^44,45^

Bacterial alginate preparations have been proposed to address this natural variability. The synthesis and molecular properties of alginate can be regulated in microbiological systems through fermentation conditions, particularly the oxygen transfer rate, which influences the G/M ratio and molecular weight.^46^ Bacterial alginate contains O-acetyl groups that affect enzymatic degradation and gel formation compared to algal alginate. Microbiological synthesis of alginate offers additional benefits, such as reduced contamination from marine biomass and greater compositional control. Despite these advantages, microbiological synthesis faces challenges, including meeting industrial application standards, achieving purification, and scaling up to large-scale production.^47^

### 2.2. Structure

SA is a natural and unbranched linear biopolymer that consists of two monomer units of α-l-guluronic acid (G unit) and β-d-mannuronic acid (M unit) linked *via* glycosidic interactions.^48^ These units are bonded in three different patterns: GG homo-polymeric units, MM homo-polymeric sequences, and MG or MG hetero-polymeric blocks, with different compositions and sequences.^35^ These structural modifications affect the physicochemical properties of SA, including mechanical strength, gelation, and viscosity. G unit exhibits ^1^C_4_ conformation, whereas M unit displays ^4^C_1_ chair conformation, which significantly affects the ability to cross-link and chain flexibility. Various analytical methods are employed to characterize the alginate due to its complex structure. Nuclear magnetic resonance (^1^H NMR) has been recognized as a promising tool for determining the M/G ratio and block distribution, thereby facilitating extensive analysis of monomer sequences. FTIR is often employed to identify the functional groups and assess chemical architecture. Compared to NMR, FTIR is less accurate for sequence analysis. Studies commonly integrate the FTIR and ^1^H NMR to elucidate the structure and composition of alginate.^49^ Size exclusion chromatography (SEC) and gel permeation chromatography (GPC) are used to evaluate the molecular weight distribution of alginate. However, the polyelectrolyte nature of alginate can affect measurement accuracy through interactions with chromatographic columns. To enhance molecular weight analysis in complex polysaccharides, advanced strategies, including asymmetrical flow field-flow fractionation integrated with light scattering, are widely utilized. Scanning electron microscopy (SEM) is often utilized to study the microstructure and morphology of SA-based compounds.

## 3. Cross linking

Crosslinking is a promising approach to enhance the adsorption performance, reusability, and structural stability of SA-based adsorbents. Sodium alginate can be crosslinked *via* ionic or physical and chemical cross-linking, owing to its numerous hydroxyl (–OH) and carboxyl (–COOH) groups.^50^ Owing to its biocompatibility and simplicity, ionic crosslinking with multivalent metal ions is the most widely used crosslinking method. However, choosing the type of crosslinking significantly affects the accessibility of active sites, swelling properties, pore features, and network structure, all of which impact the adsorption performance for various pollutant types.

### 3.1. Physical or ionic crosslinking

Ionic crosslinking is the most common strategy used for sodium alginate adsorbents, relying on the formation of the traditional “egg-box” structure *via* coordination between multivalent cations and guluronic acid segments. The egg-box model was primarily developed to explain Ca^2+^-induced gelation in alginates and remains the basic model for ionotropic gelation chemistry in polysaccharide-based systems.^51^ Several cations, such as Pb^2+^, Sr^2+^, Ca^2+^, Cu^2+^, and Ba^2+^, have been utilized as cross-linkers. Among them, calcium-crosslinked SA beads are widely used to adsorb toxic heavy-metal ions, including Cr(vi), Cu^2+^, Cd^2+^, and Pb^2+^, *via* surface complexation, electrostatic attraction, and ion exchange. For example, nanochitosan/sodium alginate (abbreviated as NCS/SA) beads were prepared employing Ca^2+^ as a crosslinking agent for the adsorption of Pb^2+^ from aqueous solution.^52^ With a maximum Langmuir monolayer capacity of Pb(ii) recorded at 178.57 mg g^−1^ at 45 °C. The experimental results showed that the adsorption data conformed to the Langmuir model, with a maximum adsorption capacity of 178.57 mg g^−1^ at 45 °C. Mechanistic investigations revealed that the exchange and electrostatic interactions were the primary mechanisms responsible for Pb^2+^ adsorption *via* the NCS/SA beads. In another study, a novel chitosan microspheres/sodium alginate hybrid bead (CSM/SA) was prepared using calcium as a cross-linking agent for the adsorption of Cr(vi) and Pb^2+^.^53^ EDX elemental mapping shows that the amount of Pb^2+^ ions adsorbed is directly proportional to the surface Ca^2+^ concentration of the CSM/SA hybrid beads. Conversely, chromium retention occurs mainly due to electrostatic attraction. The as-prepared CSM/SA composite exhibited excellent adsorption capacities of 188 mg g^−1^ for Pb^2+^ and 16 mg g^−1^ for Cr(vi). These results indicate that CSM/SA composites are cost-effective for the removal of Cr(vi) and Pb^2+^ from aqueous solutions. Similarly, alginate networks crosslinked with Zr^4+^, La^3+^, and Fe^3+^ demonstrate excellent adsorption capabilities for phosphate, thanks to robust inner-sphere complexation between Lewis acidic metal centers and phosphate species.^36^

### 3.2. Chemical crosslinking

Conversely, chemical cross-linking produces highly stable networks by generating covalent bonds between alginate chains with bifunctional agents such as epichlorohydrin, *N*,*N*′-methylene bisacrylamide, and glutaraldehyde. The chemical or covalent crosslinking significantly enhances chemical stability and mechanical strength.^54^ Furthermore, sodium alginate may form covalent bonds with amine-rich polymers such as chitosan (CS) and polyethyleneimine (PEI), thereby increasing the number of functional groups and binding sites for dyes and antibiotics. These covalently crosslinked systems exhibit improved adsorption of dyes and antibiotics *via* π–π interactions, hydrogen bonding, and electrostatic interactions. For example, Jiang *et al.*^55^ successfully prepared glutaraldehyde cross-linked eco-friendly magnetic SA beads at a specific acetic acid-to-hydrochloric acid ratio and grew sulfamic acid onto SA spheres *via* an amide process (FSSi@SA-sula) to remove moxifloxacin (MOX) and ciprofloxacin (CIP). At 318.15 K and pH 7, the highest adsorption capacities of FSSi@SA-sula were 149.57 mg g^−1^ and 365.66 mg g^−1^ for MOX and CIP, respectively. In another study, a β-cyclodextrin-immobilised SA aerogel (β-CD/NaAlg) was successfully prepared using epichlorohydrin as a crosslinking agent and evaluated as an adsorbent for tetracycline (TC).^56^ The maximum adsorption rate of β-CD/NaAlg for TC reached 70% under optimal conditions, which included an adsorbent dosage of 1.5 g L^−1^, an 8 hours contact time, and a pH of 4. The adsorption of TC by the β-CD/NaAlg composite was described by the PSO and the Freundlich isotherm models. The experimental studies revealed that the adsorption of TC onto the β-CD/NaAlg composite occurred *via* hydrogen bonding and electrostatic interaction. In conclusion, β-CD/NaAlg offers a sustainable, efficient, and eco-friendly adsorbent to eliminate TC from reclaimed water.

## 4. SA-based composite architecture

**Fig. 3:**
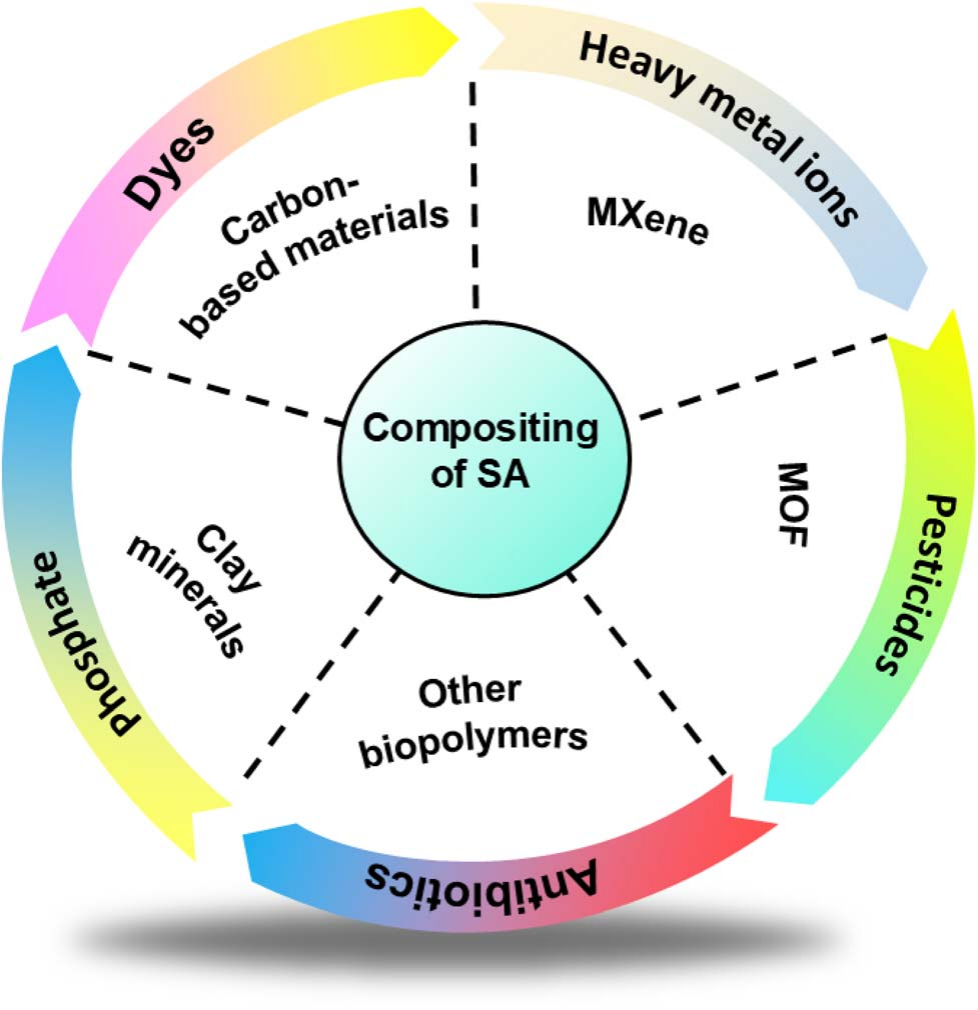
Compositing of SA with other materials for heavy metal ions, antibiotics, phosphate ions, dyes, phosphate, and pesticides adsorption.

Researchers have devoted significant effort to developing sodium alginate composite materials to enhance their adsorption properties for emerging pollutants. In this section, we comprehensively discussed the compositing of SA with carbon-based materials, clay, MXene, MOFs, and other biopolymers, as shown in Fig. 3.

### 4.1. Sodium alginate-carbon-based materials composites

Carbon-based materials, including carbon nanotubes (CNTs), biochar, and GO, are abundant in functional groups and possess high porosity, a high specific surface area, and strong mechanical strength. Stability can be improved, and adsorption sites can be exposed by combining carbon-based materials with SA.^37^Table 1 summarizes the adsorption performance of different sodium alginate-carbon-based material composites.

**Table 1:** Adsorption of water pollutants using sodium alginate-carbon-based materials composites using SA-based adsorbents

Adsorbent	Type of pollutants	Pollutant	Isotherm model	Kinetic model	Adsorption conditions	Adsorption capacity	Reusability	Ref.
SA-PAM/GO hydrogel	Heavy metal ions	Cu^2+^ and Pb^2+^	Langmuir	PSO	pH = 5, *T* = 25 °C, and agitation speed = 200 rpm	68.76 mg g^−1^ for Cu^2+^ and 240.69 mg g^−1^ for Pb^2+^	The removal rate was 80% for Cu^2+^ and 60% Pb^2+^ after the 5 cycles	92
GO–SA	Heavy metal ions	Pb^2+^, Zn^2+^, and Cd^2+^	Langmuir	PSO	*T* = 25 °C, dose = 2.8 mg, *t* = 1 min, and pH 4 for Pb^2+^, 7 for Cd^2+^, and 5 for Zn^2+^	887.21 mg g^−1^ for Pb^2+^, 161.25 mg g^−1^, for Zn^2+^, and Cd^2+^, for 139.62 mg g^−1^	—	60
SAGO aerogel	Heavy metal ions	Cu^2+^ and Pb^2+^	Langmuir	PSO	*T* = 30 °C and pH 5.5 for Pb^2+^ and 5 for Cu^2+^	267.4 mg g^−1^ for Pb^2+^ and 98.0 mg g^−1^ for Cu^2+^	—	93
SA/GO gel spheres	Heavy metal ion	Pb^2+^	Langmuir	Quasi-second-order	Pb concentration of 0–0.3 g L^−1^, *T* = 25 °C, *t* = 24 h, and ball dosage of 0.02 g	418.41 mg g^−1^	The removal rate 80% after the second cycle	94
SA-MIBC	Heavy metal ions	Thallium (Tl) and Cd	Freundlich	Tl followed PFO and Cd followed PSO	*T* = 25 °C, adsorbent dosage = 4 g, *t* = 1 min, and pH 11	36.079 mg g^−1^ for TI and 9.449 mg g^−1^ for Cd	—	95
SA@DE/CBC	Heavy metal ion	Pb^2+^	Langmuir	PSO	*T* = 298 K, adsorbent dose = 50 mg, *t* = 360 min, and pH 5	365 mg g^−1^	The adsorption rate 84% after 10 cycles	96
GO-TiO_2_/SA composite bead	Heavy metal ion	Cu^2+^	Langmuir	PSO		90.91 mg g^−1^	The adsorption rate 84.5% after 7 cycles	97
SA-M*poly(AN-*co*-ST/CNTs) composite	Heavy metal ion	Pb^2+^	Langmuir	PSO	Adsorbent dosage of 40 mg, *t* = 80 min, and pH 3	85 mg g^−1^	Maintaining 80% of its adsorption capacity after five cycles	70
CNTs@SA/PAA hydrogel	Heavy metal ion	Cu^2+^	Freundlich	PSO	Adsorbent dosage of 500 mg L^−1^, *t* = 360 min, and pH 5	358.52 mg g^−1^	—	71
KMnFC/CNT@SA	Radioactive metal ion	Uranium	Langmuir	PSO	Optimum pH was between 4 and 5, *T* = 45 °C, and adsorption equilibrium time was 24 h	437.54 mg g^−1^	89.83% of the maximum adsorption capacity after 10 cycles	98
CNT/PAM/SA	Radioactive metal ion	Uranium	Langmuir	PSO	Solid-to-liquid ratio = 0.4 g L^−1^, pH = 5.0, and uranium dosage from 25 to 300 mg L^−1^	651.39 mg g^−1^	The adsorption rate 94.56% after five cycles	99
SA/CMC-ZnONPS-AO_1_	Radioactive metal ion	Uranium	Sips	Quasi-secondary kinetic model	*T* = 298 K and pH 5	641.7 mg g^−1^	The adsorption rate 80% after five cycles	100
SA–GO composite	Radioactive metal ion	Uranium	Langmuir	PSO	Initial uranium dosage = 15 mg L^−1^, *T* = 30 °C, adsorbent = 0.1 g L^−1^, and pH = 5.0	149.76 mg L^−1^	The adsorption rate 85% after five cycles	101
La-GO/SA	Phosphate	Phosphate	Freundlich	PSO	pH = 4.0 and adsorbent dosage = 0.5 g L^−1^	34.8 mg g^−1^	—	67
La-CNT-COOH/SA	Phosphate	Phosphate	Langmuir	PSO	pH = 4.0 and phosphate dosage of 60 mg L^−1^	54.4 mg g^−1^	In five cycles, the adsorption capacity was 9.97, 9.45, 9.16, 8.86, and 8.50 mg g^−1^, respectively	75
SA-KBC-Fe/La	Phosphate	Phosphate	Langmuir	PSO	pH = 6.0, *T* = 298 K, and initial phosphate dosage = 100 mg L^−1^	46.65 mg g^−1^	Adsorption capacity remained 82% after five cycles	102
La@SA@BC	Phosphate	Phosphate	Freundlich	PSO	pH = 3, 20 mg L^−1^, and adsorbent dosage = 0.9 g L^−1^	56.5 mg g^−1^	—	103
GO/CA/SA aerogel microspheres	Dye	MB	Langmuir	PSO	—	994 mg g^−1^	The adsorption rate 90% after five cycles	104
MWCNTs-COOH/PCSAP	Dyes	MB and CV	Langmuir	PSO	pH = 9 for MB and pH = 10 for CV	662.3 mg g^−1^ for MB and 666.7 mg g^−1^ for CV	The adsorption rate 90% for MB after five cycles	105
AM–GO–SA hydrogel	Dye	CV			pH = 8.0, *T* = 30 °C, and dye = 10 mg L^−1^	100.30 mg g^−1^	The adsorption rate 90% after 10 cycles	106
Sodium alginate/graphene oxide hydrogel beads	Antibiotic	CIP	Freundlich	PSO	pH = 7.0, *T* = 30 °C, and adsorbent dosage = 480 mg	100 mg g^−1^	—	107
CNTs/L-cys@GO/SA)	Antibiotic	CIP	Langmuir-Freundlich	PSO	pH = 5.4 and *T* = 15 °C	200 mg g^−1^	—	73
GO/Ca-Alg_2_-PAM beads	Antibiotic	CIP	Langmuir	PSO	Adsorbent dosage = 0.05 g, *t* = 24, and agitation speed = 250 rpm	6.846 mg g^−1^	—	108
SA/NZVI@BC-600	Antibiotic	TC	—	—	TC concentration = 20 mg L^−1^, *t* = 90 min, and adsorbent dosage = 0.1 g L^−1^	Removal = 100%	The adsorption rate 82.46% after 10 cycles	109
SA@MRBC	Antibiotics	Tetracycline hydrochloride (TC-HCl)	Freundlich	PFO	Natural pH, adsorption temperature = 30 °C, initial TC-HCl dosage = 50 mg L^−1^, and adsorbent dosage = 2 g L^−1^	326.81 mg g^−1^	Adsorption efficiency was 67.85% after four cycles	110
GO/SA-Fe^3+^-Ca^2+^	Antibiotics	TC	Liu	PSO	*T* = 308 K, adsorbent dosage = 0.5 g L^−1^, and pH = 7	1664.05 mg g^−1^	The adsorption rate 75% after six cycles	111

#### 4.1.1. Sodium alginate–graphene oxide composites

**Fig. 4:**
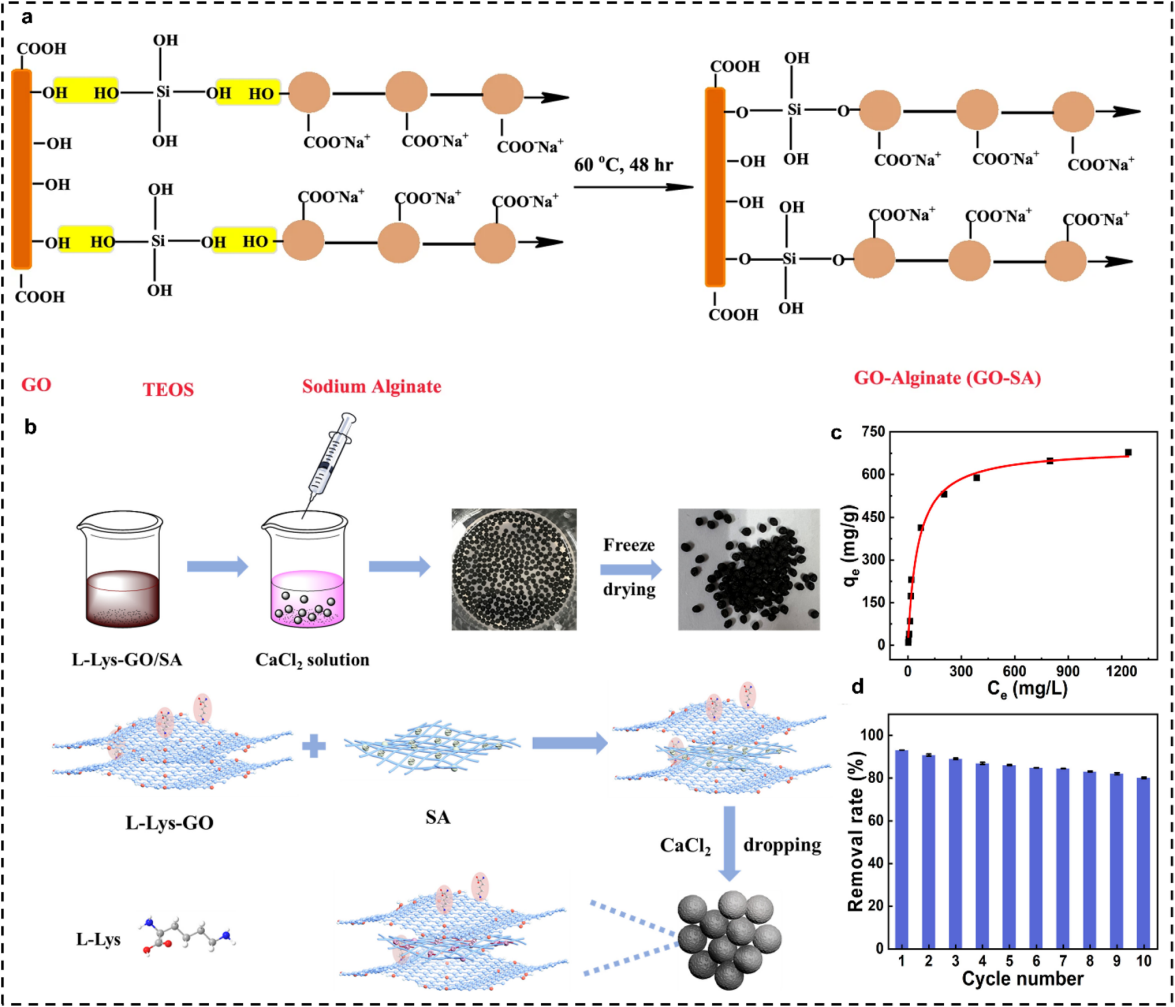
(a) Illustration of the preparation of the GO–SA adsorbent,^60^ (b) schematic illustration of the preparation for L-Lys-GO/SA beads, (c) uranium adsorption isotherm, and (d) reusability of beads in the adsorption of uranium.^61^

Graphene oxide (GO) offers advantages such as a large specific surface area, high mechanical strength, and multiple oxygen-rich functional groups. It is considered a remarkable adsorbent for pollutant removal. Due to its O-rich groups, such as COOH, –OH, –O–, and C=O, GO exhibits robust chemical and physical association with polymers. Therefore, GO can interact with hydroxyl groups of the SA *via* hydrogen bonding, thereby greatly improving the mechanical properties of SA.^57^ Recently, SA–GO composite materials have been extensively used as adsorbents. The SA–GO composite materials exhibit remarkable mechanical properties and overcome the limitations of conventional adsorbents, such as secondary pollution and poor recyclability. SA exhibits a high affinity for metal ions and a strong potential to adsorb pollutants. But sodium alginate has a drawback: inadequate mechanical characteristics.^58,59^ Thus, a composite membrane was prepared using GO/SA/1, 2-propanediamine (PDA) *via* vacuum filtration to enhance adsorption properties and structural stability. The as-prepared SA-based composite membrane was used as an adsorbent for Pb^2+^ adsorption. The composite membrane achieved a *q*_max_ of 189.25 mg g^−1^ for Pb^2+^. To further enhance adsorption performance, a novel approach was proposed to modify GO with SA using tetraethylorthosilicate (TEOS) as a binding agent (Fig. 4a).^60^ The SA–GO composite was significantly enriched with carboxylate (–COO–Na^+^) functional groups, facilitating rapid and effective interaction with metal ions. As a result, the SA–GO composite exhibited excellent *q*_max_ of 139.62, 161.25, and 887.21 mg g^−1^ for Cd^2+^, Zn^2+^, and Pb^2+^, respectively. Moreover, uranium(vi) is a non-renewable resource that has attracted researchers' attention for its reuse and recovery from wastewater. For instance, composite beads of SA and l-lysine (L-Lys) modified graphene oxides were successfully synthesized for the selective recovery of U(vi) (Fig. 4b).^61^ In addition to improving the compatibility of SA and GO, the carboxyl and amine groups in l-lysine increased the adsorption capacity of L-Lys-GO/SA composite beads for uranium. The L-Lys-GO/SA-60 beads achieved an impressive *q*_max_ of 704.22 mg g^−1^ for uranium, as exhibited in Fig. 4c. Remarkably, when ions were stimulated, the composite beads swelled, which was better for quick solid–liquid separation and low-energy U(vi) recovery. Notably, L-Lys-GO/SA-60 beads were stable after nine cycles. They exhibited an excellent adsorption efficiency of 80% after 10 cycles (Fig. 4d), highlighting their outstanding reusability and practical application prospects. In another study, new SA/PVA/GO microspheres were synthesized for the adsorption of U(vi) and Cu^2+^.^62^ The *q*_max_ were 403.78 and 247.16 mg g^−1^ for U(vi) and Cu^2+^, respectively.

**Fig. 5:**
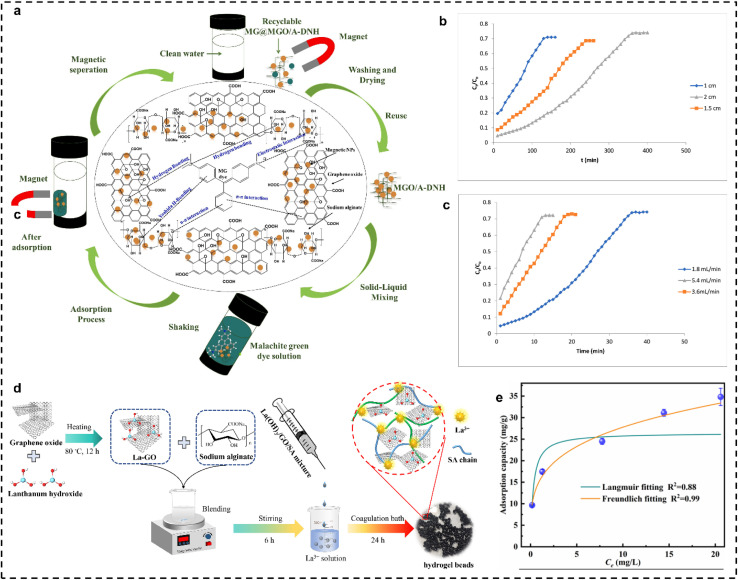
(a) Possible mechanisms for the adsorption of MG dye using MGO/A-DNH,^65^ breakthrough plots as a function of bed height (b) and flow rate (c) for 2,4-D removal,^66^ (d) synthesis of La-GO/SA composite hydrogel beads, and (e) adsorption isotherms of La-GO/SA fitted with the Freundlich and Langmuir models.

The SA–GO composite was also used to adsorb the antibiotics. For example, GO was encapsulated into an eco-friendly SA to synthesize GO–SA composite aerogel and hydrogel for the adsorption of ciprofloxacin (CIPs). Encapsulating GO in SA increased the materials' porosity, promoted π–π donor–acceptor interactions between CIP and GO, and introduced C=O groups into the composite. The *q*_max_ for GO–SA hydrogel and aerogel were 55.55 and 86.12 mg g^−1^, respectively. Recently, GO-modified κ-carrageenan (k-car)/SA gel was synthesized through calcium hardening.^63^ The introduction of GO nanosheets improved the anti-swelling characteristics and mechanical strength of the double-network hydrogel. The elastic modulus of GO-modified κ-carrageenan (k-car)/SA hydrogel is twice that of the hydrogel without GO. The double-network hydrogel achieved *q*_max_ of 197.39 and 272.18 mg g^−1^ for ofloxacin (OFL) and CIPs, respectively. Lentz *et al.*^64^ synthesized a hybrid aerogel composed of GO and SA by an external gelation approach and used it for the removal of tartrazine dye. Compared to pristine SA aerogel beads (129.64 mg g^−1^), SA–GO aerogel exhibited superior adsorption capacity (420.36 mg g^−1^) for tartrazine. A new MGO/A-DNH double-network hydrogel was prepared to adsorb malachite green (MG).^65^ This novel composite adsorbent, incorporating magnetite (M), SA, and GO, exhibits synergistic effects that enhance separation speed and adsorption performance. MGO/A-DNH hydrogel exhibited an excellent 90% removal efficiency of MG at pH 7.0 within 20 min. The higher adsorption efficiency of MGO/A-DNH adsorbent was due to hydrogen bonding, π–π/*n*–π interactions, and electrostatic interactions, favorable process conditions, and its mesoporous structure (Fig. 5a). In another study, Fe_3_O_4_, SA, and graphene composites with algal biomass were synthesized for the adsorption of 2,4-D (2,4-dichlorophenoxyacetic acid) in column and batch modes.^66^ In a batch experiment, the adsorption parameters were optimized, yielding optimal conditions of a pesticide dosage of 50 mg L^−1^, an adsorption time of 90 minutes, a temperature of 303 K, an adsorbent dosage of 0.05 g, and a pH of 2. The Na-alginate/algal bio-composite, Fe_3_O_4_/algal, graphene/algal, and H_2_SO_4_ pre-treated biomass exhibited adsorption capacities of 19.2, 21.34, 20.43, and 19.73 mg g^−1^, respectively, for 2,4-D removal. The initial dosage, bed height, and flow rate were optimized in a column experiment, and 8.55 mg g^−1^ of 2,4-D was eliminated at a 50 mg L^−1^ initial pesticide dosage, a bed height of 2 cm (Fig. 5b), and a flow rate of 5.4 mL min^−1^ (Fig. 5c). These outcomes indicated that Fe_2_O_3_, SA, and graphene composites with algal biomass are promising adsorbents for pesticide removal. Lin *et al.*^67^ prepared La-GO/SA composite beads for phosphate adsorption (Fig. 5d). The pristine graphene oxide retained a higher SSA of 2630 m^2^ g^−1^, thereby improving electrical crosslinking, surface area, and affinity for oxygen-donor materials in La-GO/SA. Surface structural and SEM studies revealed that the resultant hydrogel exhibited improved microporosity and mesoporosity. Batch mode adsorption indicated that the La-GO/SA composite showed a remarkable removal efficiency of 80% over a wide pH range of 3.0–10.0, with a *q*_max_ of 34.8 mg g^−1^ (Fig. 5e). Significantly, in the presence of interfering anions, such as HCO_3_^−^, NO_3_^−^, SO_4_^2−^, and Cl^−^, the La-GO/SA retained higher selectivity for PO_4_^3−^. The adsorption data were better fit by the PSO and the Freundlich models, suggesting a multilayer chemisorption process. The adsorption mechanisms of La-GO/SA with phosphate were proposed based on analyses including zeta potential, XRD, XPS, and FTIR. For example, the 0.5 eV in La–O and La–OH shifted after adsorption, as indicated by the XPS study, suggesting an inner–sphere complex and ligand exchange between phosphate and La(OH)_3_. Oxygen-containing groups in graphene oxides (*e.g.*, –COOH) enhance complex stability through hydrogen bonding, as evidenced by FTIR peak shifts at 1594 cm^−1^. The presence of the new O–P–O signal at 614 cm^−1^ and the typical LaPO_4_ signals at 14.55°, 20.11°, and 31.36° in the XRD spectra verified surface precipitation in La-GO/SA. The excess H^+^ ions protonate the surface of La-GO/SA under acidic conditions, leading to outer-sphere complexation as the main adsorption mechanism. Under low-pH conditions, the protonation process more readily releases –OH^2+^ ions than –OH groups from the La-GO/SA surface after phosphate uptake. Furthermore, ligand exchange was reduced, leading to a lower ΔpH. Therefore, the main mechanism was Lewis acid–base interactions, which involve the coordination of phosphate ions, such as H_2_PO_4_^−^ and HPO_4_^2−^, to La^3+^ active sites on La-GO/SA after adsorption. These composite beads exhibited excellent recyclability and aligned with green chemistry principles, highlighting their potential for industrial applications.

#### 4.1.2. Sodium alginate-carbon nanotube composites

**Fig. 6:**
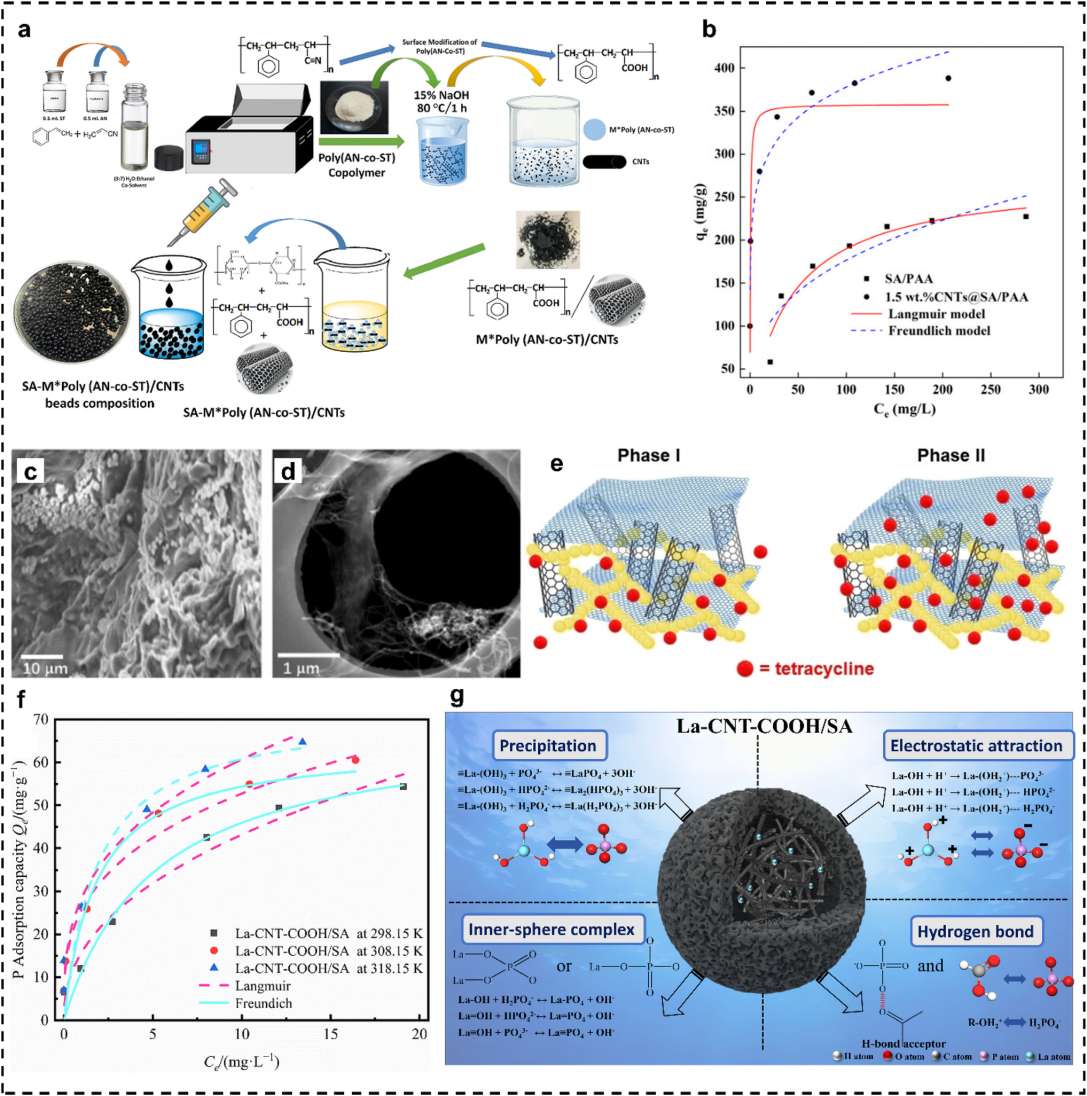
(a) Synthesis of the SA-M*poly (AN-co-ST)/CNTs composite beads,^70^ (b) adsorption isotherm plot of Cu^2+^ ions on the CNTs@SA/PAA composite hydrogels,^71^ (c) SEM and (d) TEM images of the CNT/GO/SA IPN hydrogel, (e) representation of two phases of TC adsorption by the CNT/GO/SA IPN composite hydrogel,^72^ (f) isotherm models fitted with the Freundlich and Langmuir models, and (g) suggested adsorption mechanisms of phosphate by La-CNT-COOH/SA.^75^

Similarly, CNTs have been used in various applications, including water remediation, catalysis, biosensors, and electronics, due to their remarkable properties.^68^ Owing to their superior properties, such as strong frameworks, high mechanical strength, and large surface areas, CNTs have been integrated into polymers and used as adsorbents for wastewater treatment.^69^ For example, composite beads composed of SA, CNTs, and poly(acrylonitrile-*co*-styrene) (SA-M*poly(AN-*co*-ST)/CNTs) were prepared as a promising adsorbent for Fe(ii) ions adsorption (Fig. 6a).^70^ The composite beads were synthesized by functionalizing and modifying a poly(acrylonitrile-*co*-styrene) copolymer with COOH groups. Then, SA and CNTs were combined to prepare sturdy gel beads. The as-prepared SA-based composite beads exhibited a pore size of 18.9 Å and a higher specific surface area of 127.907 m^2^ g^−1^, indicating their enhanced adsorption potential. The batch study revealed that under optimal conditions (adsorbent dosage of 40 mg, contact time of 80 min, and pH 3.0), an equilibrium adsorption capacity of 85 mg g^−1^ was achieved, with a removal efficiency of 90%. In another study, multi-wall carbon nanotubes (MWCNTs) and magnetic nanoparticles (MNPs) were combined with a mixture of SA and chitosan to prepare a magnetic composite. This innovative composite was prepared to remove iron from groundwater through adsorption. The addition of MWCNTs and MNPs provided additional binding sites and enhanced surface area. As a result, the maximum monolayer coverage of 12.43 mg g^−1^ was observed through the isotherm study. Chang *et al.*^71^ examine the removal performance of Cu^2+^ by a composite hydrogel comprising polyacrylic acid (PAA), SA, and CNTs. The characterization results indicated that the CNTs@SA/PAA hydrogel has a 3D structure with multiple pores, which serve as adsorption sites for copper ions. The FTIR study revealed that –COOH and –OH functional groups were involved in Cu^2+^ adsorption. Based on the Freundlich isotherm model, the *q*_max_ was 358.32 mg g^−1^ at room temperature (Fig. 6b). Similarly, a new interpenetrating polymer network (IPN) hydrogel was prepared as a non-selective, promising adsorbent material for antibiotics.^72^ The as-synthesized hydrogel was composed of urea-modified SA, CNTs, and GO. This IPN hydrogel comprises several active materials, including CNTs, GO, and urea-modified sodium alginate (SA) (CNT/GO/SA hydrogel). This synthetic approach began with a carbodiimide-mediated coupling between carboxylated GO and CNT, yielding a cross-linked nanocarbon network that provided a rigid scaffold with elevated porosity and abundant aromatic surfaces. An IPN hybrid gel was created by cross-linking SA within this nanocarbon network in the presence of calcium cations. The TEM and SEM analyses indicated that the resulting hydrogel exhibited high porosity and a greater surface area (Fig. 6c and d). These characteristics are advantageous for antibiotic adsorption. As a result, the CNT/GO/SA hydrogel achieved a *q*_max_ of 105.4 mg g^−1^ for tetracycline (TC) adsorption. The two-phase adsorption model of TC is depicted in Fig. 6e. The removal of TC primarily occurred on the SA in phase I due to the polar nature and hydrophilic properties of the urea-modified SA backbone. Conversely, TC adsorption on the CNT/GO/SA hydrogel was predominantly due to TC trapping and diffusion into mesopores. In another study, GO and CNTs were integrated to improve the removal performance of a traditional adsorbent material.^73^ The CNTs/L-cys@GO/SA triple-network hydrogels were prepared using cysteine (L-cys) and hydrogen peroxide. This triple-network architecture enhances the 3D porosity, increasing the number of pollutant adsorption sites and pores, thereby facilitating effective CIP adsorption. In weak alkaline media, the hydrogels exhibited *q*_max_ of 200 mg g^−1^ and 181 mg g^−1^ at 25 and 15 °C, respectively. Notably, the triple-network hydrogels exhibited superior characteristics at low temperatures. Additionally, the swelling ability, mechanical properties, and thermal stability of CNTs/L-cys@GO/SA hydrogels had improved. Makhado *et al.*^74^ prepared a (SA/p(AAc)/o-MWCNTs HNC) hydrogel for the adsorption of MB dye. At pH 8.0, the as-synthesized hydrogel displayed the highest swelling of 2265.4%. The *q*_max_ of the SA/p(AAc)/o-MWCNTs HNC was 1596.0 mg g^−1^ at 25 °C. This composite exhibited remarkable MB removal and excellent regeneration potential. Similarly, SA-CNTs-based composites are also used to remove phosphate. For example, CNTs serve as carriers to prepare a La-based SA hydrogel (La-CNT-COOH/SA) for phosphate removal from wastewater.^75^ The characterization outcomes indicated that the La(OH)_3_ nanoparticles were effectively developed onto CNT-COOH. The excellent removal performance of hydrogels was achieved at pH 4, with a *q*_max_ of 54.4 mg g^−1^ (Fig. 6f). Both chemical and physical mechanisms were involved in phosphate adsorption by La-CNT-COOH/SA, as indicated by the adsorption isotherm. Various characterizations highlighted that the loading of La(OH)_3_ on CNT-COOH was effectively involved in adsorption, alongside crosslinked lanthanum ions on SA and the presence of ample OH groups. Adsorption mechanisms by La-CNT-COOH/SA involved in-sphere complexation (La–O–P), surface precipitation, and electrostatic interactions (Fig. 6g).

#### 4.1.3. Sodium alginate-biochar composites

**Fig. 7:**
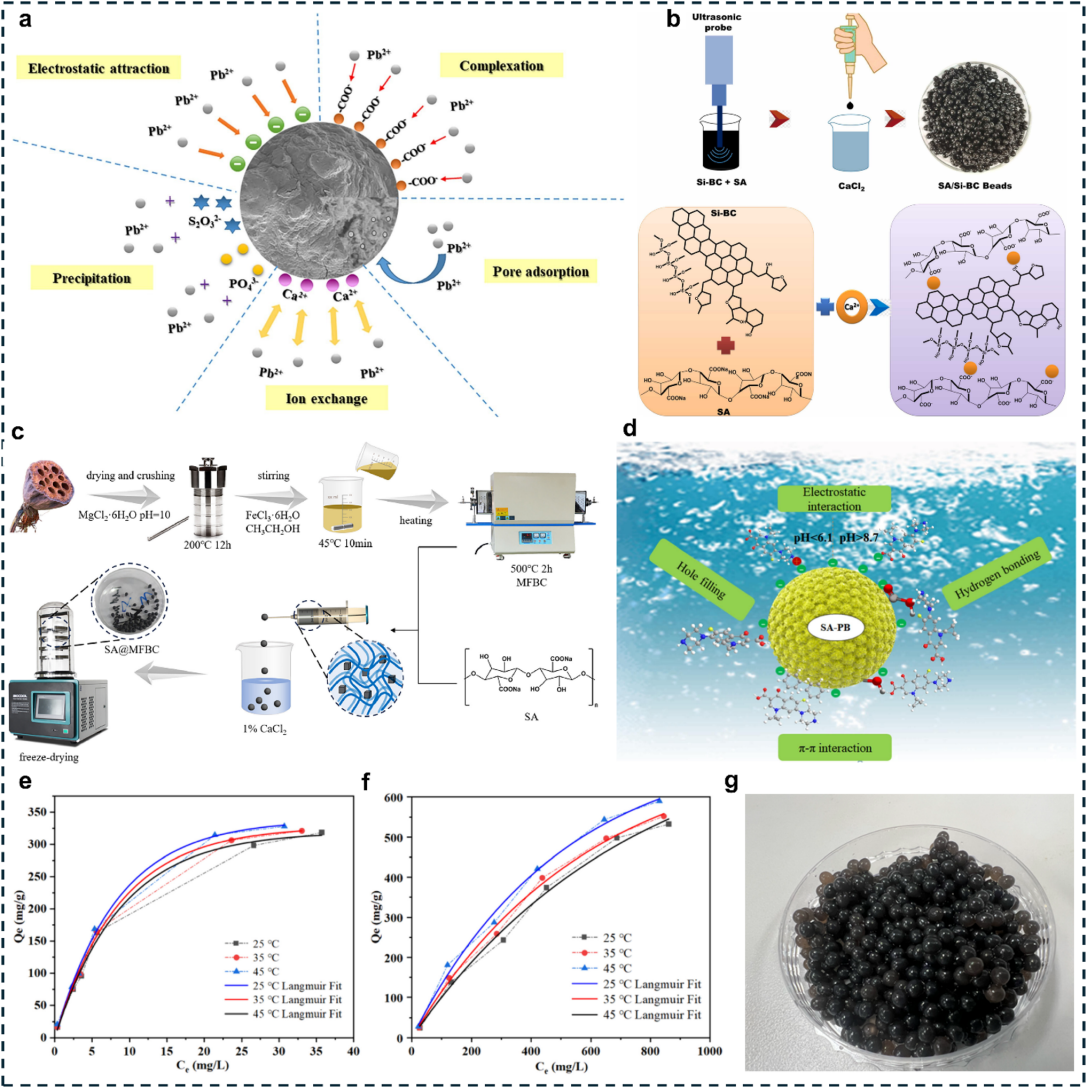
(a) Adsorption mechanism of CMGS-SA,^84^ (b) an anticipated model for the mechanism of Si-BC and SA interaction.^85^ (c) Representation for the preparation of SA@MFBC,^86^ (d) proposed adsorption mechanisms for CIP onto SA-PB in water,^88^ fitting of the Langmuir isothermal model for CR (e) and (f) MB,^90^ (g) snapshot of ABC-hydrogel.^91^

Biochar (BC), a carbon-based material derived from biowaste or biomass, has attracted significant attention as an adsorbent owing to its distinctive characteristics, including remarkable mechanical stability, high porosity, a higher specific surface area (SSA), and cost-effectiveness.^76,77^ BC has emerged as the most effective substrate for pollutant adsorption due to its cation-exchange capacity and abundant functional groups.^78,79^ The adsorption efficacy of BC is highly associated with its surface characteristics.^80^ Suitable modification of BC can increase the functional groups and active sites.^81^ Various approaches have been explored to enhance the capacity of the BC to remove pollutants. Despite these advantages, powdered BC is limited in practical applications due to challenges in recovering and separating aqueous media, leading to material loss and potential secondary pollution.^82^ To overcome this challenge, immobilizing biochar within a robust network has emerged as an effective strategy.^83^ As an eco-friendly, green, and porous material, 3D SA effectively encapsulated biochar, thereby facilitating recovery and mechanical strength. Inspired by this, a sulfuric acid-modified BC composite encapsulated with SA (CMGS-SA) was synthesized for Pb^2+^ adsorption (Fig. 7a).^84^ The characterization results exhibited the removal mechanism of CMGS-SA, which involved electrostatic attraction, precipitation, complexation, and physical adsorption in pores. The CMGS-SA exhibited excellent removal performance, achieving a *q*_max_ of 513.64 mg g^−1^. Additionally, the CMGS-SA composite retained stability across a wide pH range. It maintained greater than 80% Pb^2+^ adsorption performance after four cycles, highlighting its remarkable reusability and engineering potential. Similarly, a novel SA/Si-BC composite for Cu^2+^ adsorption.^85^ The surface of SA is negatively charged due to the presence of COOH functional groups across its backbone. Considering the surface charge and chemical composition of SA and silica-based biochar (Si-BC), a possible interaction mechanism for bead synthesis was proposed. Various functional groups of Si-BC and SA facilitated the formation of scaffolds, films, or strong gels through binding with trivalent or bivalent cations, particularly calcium ions. Ca^2+^ ions played an important role in forming cross-linking sites by attracting the Si-BC and SA chain *via* ionic interfaces. The SA biopolymer also formed hydrogen bonding with BC, as illustrated in Fig. 7b. According to the Langmuir isotherm plot, the novel SA/Si-BC composite achieved a *q*_max_ of 47.469 mg g^−1^. The adsorption isotherm results indicate that the Sips model fits copper adsorption well, as evidenced by the error analysis and the *R*^2^ value. Additionally, the error analysis and kinetic studies confirmed that the adsorption process obeyed the Avarmi model. Recently, a modified biochar aerogel bead (SA@MFBC) was prepared using SA and Mg/Fe bimetallic oxide-modified BC (MFBC) for uranium removal (Fig. 7c).^86^ Significantly, the prepared SA@MFBC-1 : 2 beads exhibited excellent thermal stability and a larger surface area. Due to abundant functional groups and porous structures, SA@MFBC-1 : 2 achieved a high adsorption capacity of 545.35 mg g^−1^. Zhang *et al.*^87^ prepared a new sodium alginate–biochar–graphene oxide composite through crosslinking for the removal of sulfadiazine (SDZ) antibiotics. The characterization results showed that the hierarchically porous structure had 2.6 times the SSA of pure SA, enhanced mechanical stability, and reinforced pore walls. The batch experiment exhibited a removal efficiency of 97.3% and a maximum adsorption capacity of 51.91 mg g^−1^ at 308 K. Fixed-bed column analysis was used to further verify practical viability, achieving 98.7% SDZ removal efficiency, with breakthrough predicted by the Thomas model. A possible mechanism was explored using FTIR and XPS analysis. The results revealed that hydrophobic partitioning, hydrogen bonding, π–π interactions, and electrostatic attraction were involved in SDZ adsorption. Significantly, the SA–BC–GO composite retained 77.7% of its adsorption capacity after 5 adsorption–desorption cycles, highlighting its outstanding reusability. By a simple phase-inversion approach, Chen, Jian, *et al.*^88^ prepared millimeter-sized sodium alginate/H_3_PO_4_ activated corncob-based biochar beads (SA-PB). The results showed that the optimal SA-to-PB mass ratio significantly enhanced the adsorption capacity for CIP, and SA-PB80 exhibited a maximum adsorption capacity of 97.10 mg g^−1^ at 298 K. Moreover, SA-PB80 demonstrated excellent reusability and biodegradability, enabling long-term application. Fig. 6d exhibited four possible interaction mechanisms, including electrostatic interaction, π–π interaction, hydrogen bonding, and hole filling, involved in the adsorption of CIPs by SA-PB. Tang *et al.*^89^ prepared composite microspheres using orange peel BC pyrolyzed at 300–900 °C and SA for the adsorption of crystal violet (CV). BC was prepared at various temperatures, and BC (pyrolyzed at 800 °C) and SA composite microspheres (BS800) exhibited superior adsorption properties with a pore size of 1.6 nm and SSA of 387.47 m^2^ g^−1^. As a result, BS800 achieved a theoretical adsorption capacity of 223.88 and 203.81 mg g^−1^ at 40 and 25 °C, respectively, as determined by the Freundlich model. The thermodynamic study verified that the adsorption of CV on the BS800 was endothermic and spontaneous. Additionally, BS800 exhibited excellent reusability, maintaining 85% of its adsorption capacity after 5 adsorption–desorption cycles, highlighting its cost-effectiveness and durability for practical applications. Recently, silver nanoparticles were synthesized using Osmanthus fragrans extract and loaded onto BC derived from barley distiller's grains shell. The C-Ag/SA/TA composite gel beads were synthesized by using the ionic crosslinking approach.^90^ C-Ag/SA/TA gel beads achieved maximum adsorption capacities of 318.06 and 166.57 for Congo red (Fig. 7e) and MB (Fig. 7f), respectively. Additionally, Ag/SA/TA gel beads showed an excellent 96.4% removal efficiency for Cr(vi). The OH groups of SA interacted with the N(CH_3_)_2_ group of the MB dye *via* hydrogen bonding. Additionally, the –COOH groups of SA were combined with the MB dye *via* electrostatic interactions. These outcomes revealed that the as-synthesized gel beads exhibited excellent adsorption capacity for metal ions and dyes. In another study, an alginate–biochar–calcium (ABC) hydrogel was prepared for phosphate removal.^91^ The BC was prepared by pyrolyzing biological sewage sludge at 600 °C and subsequently treated with NaOH to enhance its porosity and surface area. The snapshot of the ABC-hydrogel is illustrated in Fig. 7g. The as-prepared BC was then modified with calcium chloride and cross-linked with SA. These treatment methods, including crosslinking, calcium modification, and pyrolysis, effectively lower the environmental risks posed by heavy metals in biological sewage sludge. They also successfully convert sludge waste into a valuable, environmentally safe resource for practical applications. As a result, ABC-hydrogel exhibited superior adsorption performance, achieving a maximum adsorption of 252.15 mg g^−1^.

### 4.2. Sodium alginate–MXene composites

**Fig. 8:**
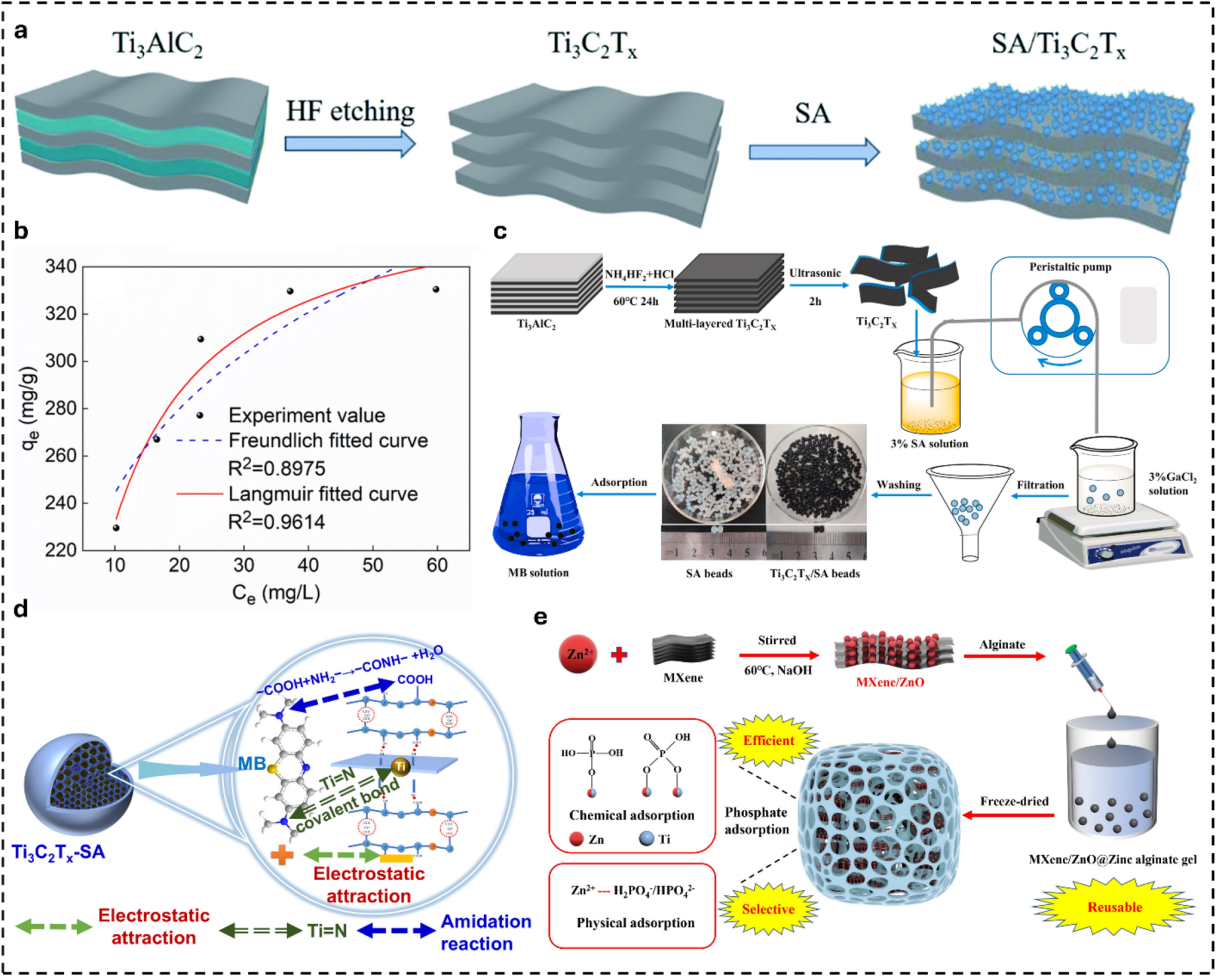
(a) Illustration for the synthesis of the MXene/alginate composites,^115^ (b) Langmuir and Freundlich fit curves,^119^ (c) schematic representation of the preparation of Ti_3_C_2_T_*x*_/SA beads for MB adsorption,^120^ (d) illustration of the possible adsorption mechanism of MB onto the Ti_3_C_2_T_*x*_@SA,^122^ and (e) representation of the preparation process and application of MXZA.^123^

MXenes, as metal nitrides, carbides, and carbonitrides, have emerged as 2D materials. The chemical formula of MXenes is M_*n*+1_X_*n*_T_*x*_, where X symbolizes N and/or C, M denotes transition metals, *n* may be 1, 2, or 3, and T_*x*_ denotes surface groups including –F, –OH, and –O.^112,113^ Due to their exceptional properties, including good catalytic performance, biocompatibility, electrical conductivity, hydrophilicity, greater reactivity, and higher specific surface area, MXenes can not only be used in the construction of biosensors, batteries, and photocatalytic materials, but also serve as efficient adsorbent materials for the adsorption of contaminants.^114^ As 2D materials, MXenes are characterized by distinct metal hydroxide sites, dual adsorption and reduction capabilities, and facile functionalization. Despite these advantages, pristine MXene exhibits a low capacity for pollutant adsorption due to its limited number of active sites. To address the challenge of limited adsorption capacity, MXene/alginate composites were prepared (Fig. 8a).^115^ Abundant carboxyl and amino groups served as chelating agents for metal ions, significantly enhancing the composites' adsorption capacity. Thus, MXene/alginate composites were used as adsorbents for the removal of Cu^2+^ and Pb^2+^ from wastewater. The MXene/alginate adsorbent increased the chelation capacity for Cu^2+^ and Pb^2+^ and promoted ion transport efficiency. As a result, the MXene/alginate adsorbent exhibited *q*_max_ of 87.6 and 382.7 mg g^−1^ for Cu^2+^ and Pb^2+^, respectively. Despite these benefits, using MXene alone is insufficient to enhance the regenerative stability of sodium alginate. A significant decrease in the removal performance of many MXene-doped composites has been identified over a few adsorption–desorption cycles.^116^ Polyaniline (PANI) is a promising alternative for addressing these challenges owing to its remarkable properties, including regenerative capabilities, facile synthesis, cost-effectiveness, and environmental resilience. Thus, an innovative composite (PANI@SA-SNM) was prepared by combining amine/sulfur-modified MXene (SNM) with *in situ* polymerized SA and PANI for the adsorption of Hg^2+^ and Cu^2+^.^117^ The resulting composite achieved *q*_max_ values of 352.76 and 255.81 mg g^−1^ for Hg^2+^ and Cu^2+^, respectively. The adsorption and desorption performance of the PANI@SA-SNM composite for Hg^2+^ and other metal ions was enhanced over eight cycles, underscoring its exceptional reusability and practical applicability. XPS analysis was performed to better understand the possible adsorption mechanisms of Hg^2+^ and Cu^2+^ on the PANI@SA-SNM composite. The Hg 4f and Cu 2p signals were identified in the XPS survey spectrum, verifying the adsorption of Cu^2+^ and Hg^2+^ on the PANI@SA-SNM composite. At the same time, the reduction in the Ca2p peak further verified that ion exchange occurred during adsorption. Zhou *et al.*^118^ prepared a 3D macroscopic MPAC aerogel using oxidized SA, MXene@polydopamine, and leather collagen for the adsorption of heavy metal ions. The Freundlich isotherm model exhibited the lowest *χ*^2^ values (20.8116, 11.3378, and 15.6383), RMSE values (1.8624, 1.3746, and 1.6144), and a superior correlation coefficient (*R*^2^ > 0.9809). The MPAC adsorbent achieved a *q*_max_ of 114.74 mg g^−1^ for chromium at 20 °C. Thermodynamic studies revealed that the removal of chromium by MPAC adsorbent was an endothermic and spontaneous process. Luo and his colleagues prepared an ultrathin, nanolayered adsorbent by combining TiVCT_*x*_ with SA *via* a simple freeze–drying strategy.^119^ TiVCT_x_ achieved an excellent *q*_max_ of 336 mg g^−1^ for U(vi) from high-concentration wastewater (Fig. 8b). The sorption mechanism was extensively studied, indicating that the electrostatic removal of uranium by TiVCT_*x*_. TiVCT_*x*_ exhibited better removal performance and greater resistance to co-existing ion interference, indicating its promise for uranium adsorption in seawater.

Recently, composite beads were prepared *via* ionic crosslinking using SA and Ti_3_C_2_T_*x*_ for the adsorption of MB dye (Fig. 8c).^120^ Ti_3_C_2_T_*x*_ MXene was tightly bound to the SA, significantly reducing the loss of the composite adsorbent and enhancing adsorption, as evidenced by XRD, FTIR, and SEM. The outcomes indicated that a Ti_3_C_2_T_*x*_/SA mass ratio of 30% was suitable for MB adsorption. The Ti_3_C_2_T_*x*_/SA-30% achieved a *q*_max_ of 92.17 mg g^−1^. The MB adsorption rate on Ti_3_C_2_T_*x*_/SA-30% was retained at 81.36% after three adsorption–desorption cycles. Although multivalent cation-based crosslinking enhances the physicochemical stability of the composite adsorbent, MXene nanosheets may be poorly dispersed or buried within the SA polymer network, potentially reducing adsorption efficiency. To overcome this issue, a series of SA/MXene nanofiber membranes (NMs) was synthesized using Ca^2+^-mediated crosslinking and electrospinning.^121^ The SA/MXene NMs exhibited a *q*_max_ of 440 mg g^−1^, which was significantly higher than that of pristine MXene, SA/MXene beads, and electrospun SA NMs. This outstanding adsorption performance was attributed to the 3D fibrous nanostructure, which facilitates facile access of MB to the SA/MXene binding sites. Moreover, the SA/MXene NMs exhibited outstanding reusability. After 10 cycles, both the MB removal rate and the adsorption capacity were maintained at 95% of those of the fresh sample, demonstrating that the electrospinning approach has remarkable potential to synthesize biomass-based materials with high efficiency. In another study, a spherical foam composite was prepared using SA and Ti_3_C_2_T_*x*_ MXene for the MB adsorption.^122^ Four Ti_3_C_2_T_*x*_@SA spherical foams with lamellar and honeycomb-like structures were prepared by varying the volume of the Ti_3_C_2_T_*x*_ solution and compared in terms of MB adsorption performance. Lamellar and honeycomb-like spherical foams exhibited higher removal performance. The *q*_max_ of the Ti_3_C_2_T_*x*_@SA honeycomb-like porous foam for MB was 969 mg g^−1^. Additionally, the adsorption of methylene blue by Ti_3_C_2_T_*x*_@SA honeycomb-like foam was endothermic, spontaneous, and aligned with the monolayer adsorption model, mainly driven by the combined action of amidation and electrostatic adsorption (Fig. 8d). Liao and his colleagues used SA to integrate MXene/ZnO composites to synthesize a 3D MXZA hydrogel *via* an ion-crosslinking strategy (Fig. 8e).^123^ They employed it as a highly reusable, selective, and efficient phosphate adsorbent. The *q*_max_ of MXZA for PO_4_^3−^ was 86.67 mg g^−1^. Additionally, MXZA exhibited outstanding selectivity toward complex ions, including SO_4_^2−^, NO_3_^−^, and Cl^−^. In another study, SA/MX/CFO was developed using SA, CoFe_2_O_4_, and MXene for the removal of CIP and Cu^2+^ with a magnetic field.^124^ The SA/MX/CFO exhibited outstanding mechanical characteristics, with an elastic modulus of 2.23 MPa and a fracture stress of 1.64 MPa. The experimental results indicated that the adsorption capacity of CIP increased by 24.2%. The adsorption performance of several sodium alginate–MXene composites is exhibited in Table 2.

**Table 2:** Adsorption of water pollutants using sodium alginate–MXene composites using SA-based adsorbents

Adsorbent	Type of pollutants	Pollutant	Isotherm model	Kinetic model	Adsorption conditions	Adsorption capacity	Reusability	Ref.
MXene/alginate composites	Heavy metal ions	Pb^2+^ and Cu^2+^	Langmuir	PSO	pH ≥ 5 and adsorbent dose = 50 mg	382.7 mg g^−1^ for Pb^2+^ and 87.6 mg g^−1^ for Cu^2+^	The adsorption loss rates of Pb^2+^ and Cu^2+^ were 8.9% and 5.4% after ten cycles	115
PANI@SA-SNM	Heavy metal ions	Cu^2+^ and Hg^2+^	Langmuir	PSO	pH = 4.0, *T* = 303.15 K and adsorbent dose = 0.02 g/50 mL	352.76 mg g^−1^ for Hg^2+^ and 255.81 mg g^−1^ for Cu^2+^	The desorption rates were 91% through eight cycles	117
MPAC composite aerogel	Heavy metal ion	Cr(vi)	Freundlich	Two-level dynamics model	*T* = 293 K and pH = 1	144.74 mg g^−1^	The removal efficiency was 70.4% after the 5 cycles	118
MXZA	Phosphate	Phosphate	Langmuir	PSO	*H* = 4.0, *T* = 298 K and phosphate concentration = 20 mg L^−1^	86.67 mg g^−1^	—	123
MX-ZrSA	Phosphate	Phosphate	Freundlich and optimized Langmuir models	PSO and intraparticle diffusion	pH = 3.03 and adsorbent dose = 500 mg L^−1^	492.55 mg g^−1^	The adsorption capacity decreased was 70.4% after the 5 cycles	125
Ti_3_C_2_T_*x*_/SA beads	Dye	MB	Langmuir	PSO	pH = 7.0 and *T* = 298 K	92.17 mg g^−1^	Adsorption efficiency was 81.36% after three cycles	120
SA/MXene nanofiber membrane	Dye	MB	Langmuir	PSO	pH = 7.0, *T* = 298 K, *t* = 24 h, and adsorbent dose = 3 mg	440 mg g^−1^		121

### 4.3. Sodium alginate–MOFs composites

Metal–organic frameworks (MOFs) have gained significant attention due to their controllable topology, fine structures, and high porosity. MOFs are considered promising materials for applications in energy storage, adsorption, gas separation, drug delivery, and heterogeneous catalysis.^126,127^ Currently, various MOFs are used as adsorbents for pollutant removal and for the recovery of noble metals.^128^ However, few MOFs exhibit significant issues. These materials have limited application in wastewater treatment because they become structurally unstable under wet conditions. A critical challenge in employing MOFs for wastewater treatment is the substantial loss of adsorbent during regeneration and separation from the liquid phase. It is considered that these obstacles may be addressed by encapsulating metal–organic frameworks within alginate hydrogel particles. Inspired by this concept, MOF-doped alginate beads (MOFs@ABs) were prepared to adsorb Cr(vi).^129^ The results indicated that UiO-66 (0.8 mL)@ABs achieved the highest removal rate of 98% for Cr(vi). However, the dosage of the positively charged MOF was essential for Cr removal. Furthermore, pH altered the surface charges of both the adsorbate and the adsorbent, thereby enhancing Cr(vi) adsorption. The UiO-66@Abs indicated a satisfactory regeneration ability of 82% for Cr(vi). ZIF-8, a MOF that has considerable advantages such as high porosity, multiple active sites, and a larger specific surface area, has broad applications in environmental treatment. Despite these advantages, powder ZIF-8 is prone to agglomeration, which makes it difficult to separate from aqueous media and thereby restricts its practical application in water remediation. To address this issue, SA was selected as the gel matrix, and CaCl_2_ as a cross-linking agent to prepare ZIF-8/SA.^130^ The results indicated that the saturated adsorption capacities of the as-prepared composites were 101.86 and 179.86 mg g^−1^ for the Mn–Cu–Cd composite pollution solution and the single Mn^2+^ pollution solution, respectively. The XPS, FTIR, and SEM-EDS analysis indicated that the adsorption mechanism of ZIF-8/SA involved electrostatic attraction, coordination of amino/hydroxyl/carboxyl groups, and ion exchange. In another study, ZIF-9 was developed as the PVA/SA hydrogel for Cu^2+^ adsorption.^131^ This composite was developed to improve the poor mechanical strength of SA hydrogels and to mitigate the issue of powdered MOF in practical applications. The experimental outcomes indicated that the –COOH and –OH functional groups of the composite participated in the Cu uptake, and physical adsorption, cation exchange, and complexation were key adsorption mechanisms. The PVA/SA@ZIF-9 composite exhibited a *q*_max_ of 98.98 mg g^−1^ for Cu^2+^.

**Fig. 9:**
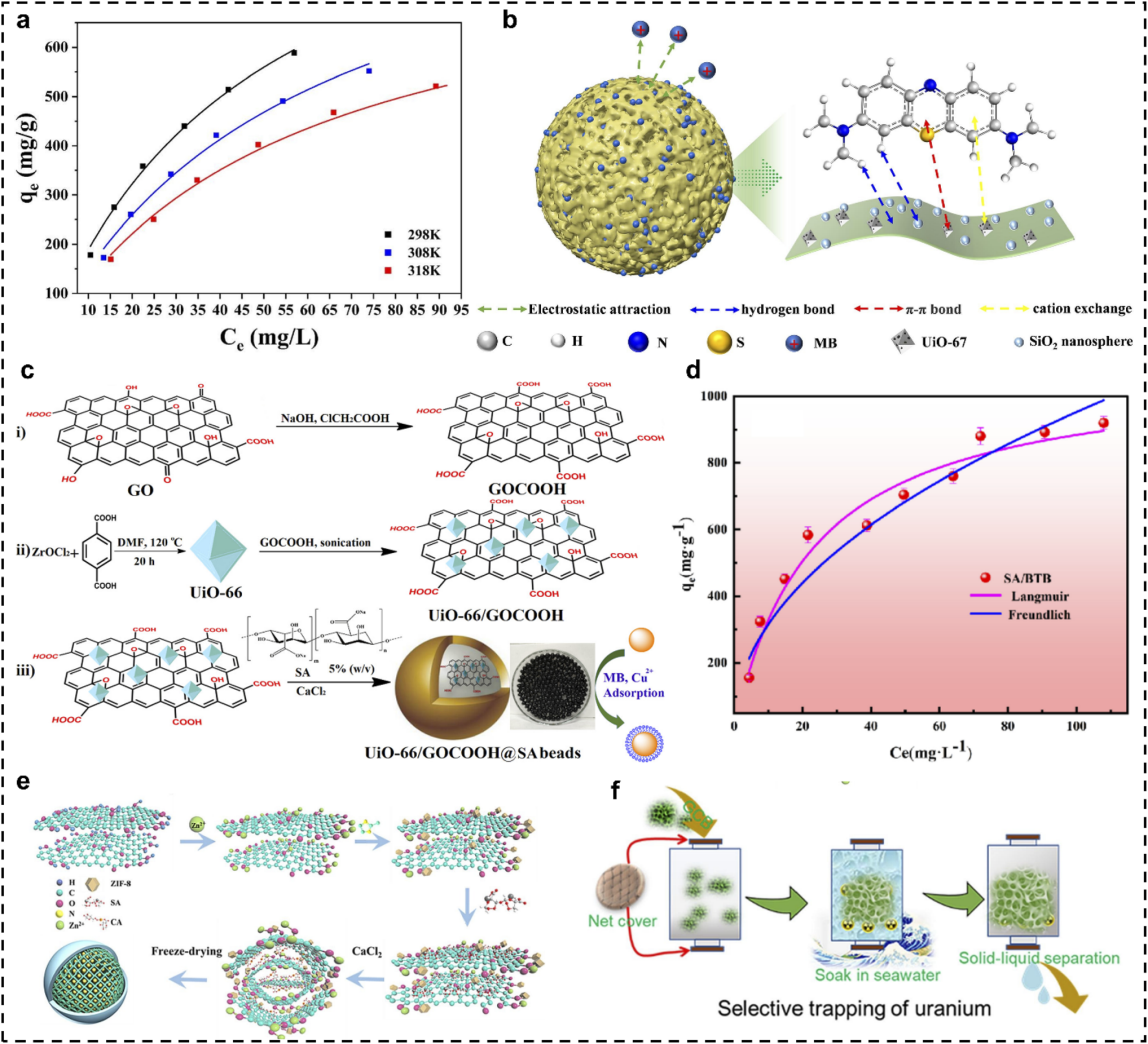
(a) Isotherm adsorption model of removal of MB dye using SA@SiO_2_@UiO-67 beads, (b) illustration of possible adsorption mechanism of MB onto the SA@SiO_2_@UiO-67 aerogel surface,^132^ (c) illustration of the synthesis of the UiO-66/GOCOOH@SA composite,^134^ (d) isothermal model of adsorption of U(vi) using SA/BTB composite,^135^ (e) schematic illustration of the synthesis process of GO/ZIF-8@SA, and (f) selective trapping of GO/ZIF-8@SA composite to U(vi).^136^

Zhou *et al.* prepared a MOF-based dye adsorbent, SA@SiO_2_@UiO-67 beads, *via* a hydrothermal strategy, using a zirconium organic skeleton as the nanocrystals for *in situ* growth of MOF and SA@SiO_2_ as the carrier.^132^ The experimental result indicated that the SA@SiO_2_@UiO-67 adsorbent exhibited a *q*_max_ of 1094.04 mg g^−1^ for MB dye at 298 K (Fig. 9a). The kinetic study revealed that the methylene blue dye was physically adsorbed onto the SA@SiO_2_@UiO-67 composite. The as-prepared composite achieved an MB removal efficiency of 71.64% after six cycles. The characterization studies revealed that the combined effects of various mechanisms, including diffusive mass transfer, hydrogen bonding, ion exchange, and electrostatic interactions, facilitated the removal of MB dyes from the aerogel surface (Fig. 9b). Phu *et al.*^133^ prepared hierarchical porous composite microgels (SPZ microgels) using ZIF-8, polyvinyl alcohol (PVA), and SA through microfluidic technology. The introduction of ZIF-8 led to the formation of a porous architecture within the microgels, thereby significantly enhancing their dye adsorption performance. The as-prepared SPZ microgels exhibited outstanding removal capacities for cationic and ionic dyes. The SPZ microgels exhibited remarkable removal capacities of 210 and 180 mg g^−1^ for MB and methyl orange (MO), respectively. The adsorption of both MB and MO was promoted by physical entrapment within the porous network, hydrogen bonding, and electrostatic interactions. In a separate study, a novel MOF-based bead adsorbent was prepared by combining GOCOOH and UiO-66 MOF with SA for the removal of Cu^2+^ ions and MB (Fig. 9c).^134^ The introduction of GOCOOH and MOF significantly improved their adsorption performance for Cu^2+^ ions and MB dye, exhibiting *q*_max_ values of 343.49 and 490.72 mg g^−1^, respectively. The adsorption of both Cu^2+^ ions and MB dyes obeyed the Freundlich and the PSO models. Two new signals at 400.11 and 165.18 eV were identified in the XPS survey spectrum of the composite beads after MB adsorption, corresponding to nitrogen and sulfur in MB. Furthermore, the O 1s spectra before and after MB uptake exhibited a shift from 532.92 to 532.86 eV, confirming MB's uptake onto the composite beads. Additionally, the N 1s spectrum revealed a distinct peak at 399.37 eV, indicating the presence of –N(CH_3_)_2_ and confirming MB uptake onto the composite beads. MB dye was adsorbed onto the composite *via* electrostatic attraction between the negatively charged beads and the positively charged dye. In addition to electrostatic attraction, van der Waals forces and *n*–π/π–π interactions also played significant roles in MB adsorption onto composite beads. Similarly, a new distinct peak at 934.5 eV for Cu 2p was observed in the XPS survey spectrum after Cu^2+^ adsorption. Furthermore, the observed reduction in the Ca 2p peak intensity indicates that Ca^2+^ is partially replaced by Cu^2+^. This suggests that Cu^2+^ uptake onto SA proceeds through an ion-exchange mechanism. Additionally, the high-resolution spectrum of Cu 2p showed characteristic peaks at 952.9 and 933.1 eV for the 2p_1/2_ and 2p_3/2_ states, respectively. This suggests the formation of –O–Cu^2+^ or –OH–Cu^2+^ complexes. Additionally, electrostatic interactions play a significant role in Cu^2+^ adsorption, as cationic Cu^2+^ is strongly attracted to the O-containing functional groups on the composite bead. Mao *et al.*^135^ designed a novel bio-composite of SA doped with a hydrogen-bonded organic framework to enhance SA's uranium adsorption performance. The incorporation of SA and hydrogen-bonded MOF provided outstanding thermal stability and mechanical strength. The SA/MOF composite exhibited superior adsorption performance of 1087 mg g^−1^ (Fig. 9d) and recyclability. The adsorption of U(vi) onto the SA/MOF followed the PSO model, revealing that the chemosorption mechanism was dominated by surface complexation. The thermodynamic study revealed that uranium uptake was spontaneous and endothermic. In another study, a 3D carbon architecture composite material (GO/ZIF-8@SA) was synthesized using SA, GO, and ZIF-8 (Fig. 9e).^136^ The results indicated that the *q*_max_ of GO/ZIF-8@SA for U(vi) was 1897 mg g^−1^ in uranium-spiked seawater. The removal mechanism was studied using FTIR and XRD. The outcomes indicated that ligand and ion coordination were involved in uranium capture. Additionally, the greater the amount of uranium adsorbed by composite spheres, the greater the volume expansion. A simple device was constructed to use GO/ZIF-8@SA spheres to adsorb U(vi) (Fig. 9f). The composite spheres increased in size after adsorbing U(vi) and were retained in the bottle during filtration. This straightforward approach not only significantly reduced separation time but also improved separation efficiency and lowered separation cost. Wang and his coworkers synthesized a novel SA@Fe@MOF-Al adsorbent for H_2_PO_4_^−^ adsorption from wastewater.^137^ The SA@Fe@MOF-Al composite exhibited a *q*_max_ of 103.09 mg g^−1^. However, a thermodynamic study revealed that the removal of H_2_PO_4_^−^ from wastewater using SA@Fe@MOF-Al composite was spontaneous and exothermic. The adsorption of H_2_PO_4_^−^ followed the Langmuir isotherm model and the PSO kinetic model. Guesmi *et al.*^138^ combined a novel MSe-MOF into alginate/chitosan beads (MSCA) for efficient removal of CIP. The characterization results confirmed the formation of a porous structure with a high SSA of 420 m^2^ g^−1^, thereby improving pollutant adsorption. The MSCA exhibited a *q*_max_ of 440 mg g^−1^ for CIP. Table 3 displays the adsorption performance of various sodium alginate-MOFs composites.

**Table 3:** Adsorption of water pollutants using sodium alginate–MOF composites using SA-based adsorbents

Adsorbent	Type of pollutants	Pollutant	Isotherm model	Kinetic model	Adsorption conditions	Adsorption capacity	Reusability	Ref.
Cys@UiO-67-bpy aerogel beads	Heavy metal ions	Cd^2+^, Cu^2+^, and Pb^2+^	Sips	PSO	pH = 5.5 and *T* = 318 K for Cd^2+^, pH = 5.5 and *T* = 318 K for Cu^2+^, and pH = 5.0 and *T* = 318 K	296.2 mg g^−1^ for Cd^2+^, 326.4 mg g^−1^ for Cu^2+^, and 661.2 mg g^−1^ for Pb^2+^	91.20% for Cd^2+^, 90.42% for Cu^2+^, and 77.9% for Pb^2+^	139
MOF-5/PNaSS/SA hydrogel	Heavy metal ion	Pb^2+^	Langmuir	PSO	pH = 6 and adsorbent dosage = 0.5 g L^−1^	189 mg g^−1^	Pb(ii) adsorption decreased slightly from 189 mg g^−1^ to 181 mg g^−1^	140
D-MOF-801/SA aerogel beads	Heavy metal ion	Pb^2+^	Sips	PSO	pH = 4 and *T* = 298 K	375.48 mg g^−1^	The removal efficiency of ∼90.23% after 5 cycles	141
SA-ME@ZIF-67	Heavy metal ion	Pb^2+^	Langmuir	PSO	pH = 5 and *T* = 308.15 K	634.99 mg g^−1^	After five cycles, the removal efficiency of was 85%	142
Sulfonic-functionalized A-U6S composite	Heavy metal ion	Pb^2+^	Langmuir	PSO	pH = 5, 40 mL of the Pb^2+^ solution, *t* = 24 h, and adsorbent dosage = 0.01 g	400.5 mg g^−1^	The removal rate 94.2% after the 5 cycles	143
PAM/SA/PAA@ZIF-67	Radioactive metal ion	Uranium	Langmuir	PSO	pH = 5, *t* = 25 h, and *T* = 25 °C	500.0 mg g^−1^	The adsorption rate 90.79% after 8 cycles	144
MNPs-SA@Cu MOF	Radioactive metal ions	Uranium and thorium	Langmuir	PSO	pH = 6, 10 mL of the uranium solution, 50 mL of the thorium solution, and adsorbent dosage = 6 mg	454.54 mg g^−1^ for uranium and 434.78 mg g^−1^ for thorium	The removal efficiencies of U(vi) and Th(iv) were 92% and 89.25%, respectively, after 10 cycles	145
UiO-66-NH_2_/SA@PEI	Phosphate	Phosphate	Freundlich	PSO	pH = 2, *T* = 65 °C, and adsorbent dosage = 0.05 g	68.75 mg g^−1^	Adsorption rate remained at 99% after five cycles	146
MIL101/Alg beads	Phosphate	Phosphate	Langmuir	PSO	pH = 6, phosphate concentration 300 mg L^−1^, and adsorbent dosage = 2.5 g L^−1^	109.5 mg g^−1^	MIL101/Alg maintained the removal efficiency of >80%	147
La–Al MOF/Zr-Alg macroscopic beads	Phosphate	Phosphate	Liu isotherm model	—	pH = 3 and *T* = 298 K	105.83 mg g^−1^	The adsorption rate remained at 80% after five cycles	148
APBCLOF composite	Phosphate	Phosphate	Freundlich	PSO	pH = 7 and *t* = 30 min	47.5 mg g^−1^	70% phosphate removal efficiency upto 5 cycles	149
Co@Cu–MOF/alginate beads	Antibiotic	CIP	Freundlich	PSO	pH = 9, *t* = 45 min, and adsorbent dosage = 0.17 g	95.75 mg g^−1^	The adsorption efficiency remained at 90% after four cycles	150
ZIF-67/MIL-88A@Alg@PDA beads	Antibiotic	Doxycycline (DOX)	Freundlich	PSO	pH = 7, *T* = 25 °C, and adsorbent dosage = 0.02 g	384.61 ± 5.08 mg g^−1^	After eight cycles, the removal rate decreased by 15.42%	151
Alg@MOF-rGO hydrogel beads	Antibiotics	TC and CIP	Langmuir	PSO	pH = 7 and *T* = 40 °C	43.76 mg g^−1^ for TC and 40.76 mg g^−1^ for CIP	—	152
SA/ATP/ZIF-8	Antibiotic	Norfloxacin (NOR)	Langmuir–Freundlich	PSO	pH = 5	1360.67 mg g^−1^	The adsorption capacity remained over 77% after five cycles	153
(SA-*g*-P3AP@MOF(Fe)/Ag)	Antibiotic	Neomycin	Freundlich	PSO	pH = 7, *t* = 30-min, adsorbent dosage = 5 mg, and *T* = 25 °C	625 mg g^−1^	After three cycles, the adsorption efficiency was 79%	154
SA@ZIF-8 composite	Dye	Malachite green (MG)	Freundlich	PSO	pH = 6, *T* = 308 K, and *t* = 120 min	69.97 mg g^−1^	—	155
UiO-66/GOCOOH@SA	Dye		Freundlich	PSO	pH = 11, adsorbent dosage = 0.025 g	490.72 mg g^−1^	Removal efficiency remained 87% after five cycles	134
CA-MIL-53-AC	Pesticides	DDT	Langmuir	PFO	pH = 3 = 11, *T* = 40 °C, and initial dosage of DDT = 5 mg L^−1^	5.29 mg g^−1^	Removal efficiency remained 87% after five cycles	156

### 4.4. Sodium alginate–clay minerals composites

**Fig. 10:**
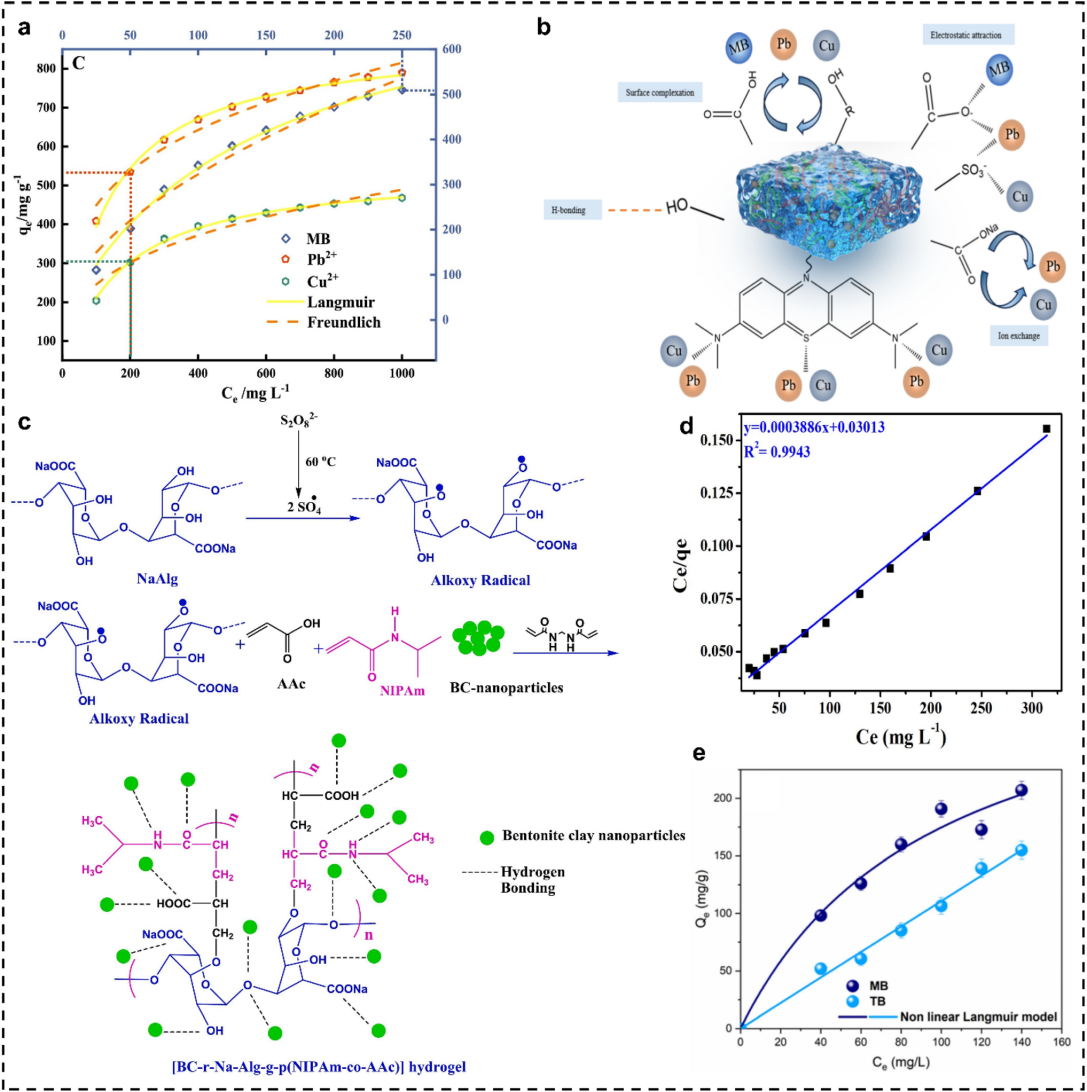
(a) Isothermal model of adsorption of Pb^2+^, Cu^2+^, and MB using SA/CMC/SKL composite, (b) the suggested removal mechanism for Pb^2+^, Cu^2+^, and MB,^161^ (c) an illustration of the synthesis process of [BC-*r*-Na-Alg-*g*-p(NIPAm-*co*-AAc) composite, (d) Langmuir plots for MG adsorption onto the [BC-*r*-Na-Alg-*g*-p(NIPAm-*co*-AAc)] adsorbent,^162^ and (e) Langmuir plot for the removal of TB and MB using ALG@Mmt@HTAB beads.^164^

Clay minerals, including bentonite (BT), illite, kaolin (KN), and montmorillonite (Mt), are broadly used as adsorbents owing to their high charge densities, large surface areas, and layered structures.^157,158^ The occurrence of various functional groups on clay surfaces facilitates pollutant binding. Recently, significant attention has been attracted to combining biopolymers with clay minerals to improve adsorption performance.^159,160^ For example, an SA/CMC/SKL hydrogel was successfully developed using sulfuric acid-modified kaolin (SKL), SA, and CMC.^161^ The resultant SA/CMC/SKL hydrogel was used to remove Cu^2+^, Pb^2+^, and MB. Based on the isotherm and kinetic analyses, the adsorption data were better described by the Langmuir and PSO models. The SA/CMC/SKL hydrogel achieved maximum capacities of 543.50, 879.84, and 805.16 for Cu^2+^, Pb^2+^, and MB dye, respectively (Fig. 10a). XPS study confirmed that electrostatic attraction and ion exchange primarily contributed to the adsorption of Cu^2+^, Pb^2+^, and MB dye (Fig. 10b). Shah *et al.*^162^ prepared a novel adsorbent, BC-*r*-Na-Alg-*g*-p(NIPAm-*co*-AAc), using poly(*N*-isopropyl acrylamide-*co*-acrylic acid), SA, and BT to remove methylene green (MG), as illustrated in Fig. 10c. The swelling capability of resulting hydrogel composite was enhanced with a rise in pH value. The experimental outcomes indicated that the adsorption data were better fitted to the PSO kinetic model and the Langmuir isotherm, suggesting chemisorption as the dominant interaction. Overall, the simple synthesis, outstanding swelling (9664%), remarkable recyclability (6 cycles), and high adsorption capacity (2573 mg g^−1^) (Fig. 10d), rendered the resultant adsorbent a promising alternative for industrial applications. In ecological remediation, montmorillonite is commonly combined with biopolymer networks, such as SA, to develop a composite with ion-exchange properties and enhanced adsorption capacity.^163^ This combination hinders the loss of clay particles, protects active sites from deactivation, and retains the functional surface area. However, the challenges, including the necessity for regeneration, limited selectivity, and possible aggregation, highlight the importance of optimizing clay modification and composite structure to improve performance for practical applications. Given these challenges and the properties of clay-based adsorbents, synthesizing a composite that combines the structural benefits of biopolymers, such as SA, with the high adsorption performance of Mt has emerged as a promising approach for pollutant removal. For example, a composite hydrogel (ALG@Mmt@HTAB) was prepared using hexadecyltrimethylammonium bromide modified Mt and SA to adsorb trypan blue (TB) and MB.^164^ The ALG@Mmt@HTAB adsorbent displayed an internal layered structure with nano-sized features, and the modified Mt enhanced the porosity of the composite. The ALG@Mmt@HTAB hydrogel exhibited adsorption capacities of 482.22 and 345.88 for TB and MB, respectively (Fig. 10e). However, challenges persist in effectively removing uranium due to competition among negatively charged sites at the clay end face, on the surface, and in interlayer spaces, as well as limited mineralization capacity. To address this issue, the BT, nontronite (Nt), Mt, polyethylene (PE), and SA were used to synthesize three composite hydrogel adsorbents (abbreviated as BT/PE-@SA, Nt/PE-@SA, and Mt/PE-@SA).^165^ The resultant composite hydrogels were used to remove U(vi) from aqueous solution. The composite hydrogels exhibited selective removal and mineralization of U(vi) due to the combined effects of PE and SA groups. The Nt/PE-@SA hydrogels exhibited higher *q*_max_ than the BT/PE-@SA and Mt/PE-@SA hydrogels, achieving an adsorption capacity of 133.3 mg g^−1^ at pH 5.5. The selectivity analysis indicated that the Mt/PE-@SA had abundant functional groups, including –COO and –OH, which contributed to its high adsorption selectivity and affinity for U(vi). Chang *et al.*^166^ synthesized composite beads with a 1 : 1 or 2 : 1 SA-to-Mt ratio for TC adsorption. The maximum TC adsorption capacities were 689 and 745 mg g^−1^ for 1 : 1- and 2 : 1-Mt/SA, respectively. Hydrogen bonding, electrostatic attraction, and cation exchange contributed to TC adsorption. The introduction of a minimal amount of TC into the beads' internal cavities led to TC intercalation within the Mt interlayers, thereby enhancing TC adsorption by the Mt/SA composite beads. Table 4 highlights the adsorption performance of various sodium alginate–clay minerals composites.

**Table 4:** Adsorption of water pollutants using sodium alginate–clay composites using SA-based adsorbents

Adsorbent	Type of pollutants	Pollutant	Isotherm model	Kinetic model	Adsorption conditions	Adsorption capacity	Reusability	Ref.
SEP@PEI/SA	Heavy metal ion	Pb^2+^	Langmuir	PSO	*T* = 25 °C, *t* = 24 h, pH = 5.5, and adsorbent dosage = 20.0 mg	1094.86 mg g^−1^	—	167
Alginate/kaolin beads	Heavy metal ion	Cu^2+^	Langmuir	PSO	pH = 3–4 and Cu^2+^ concentration = 1 mg L^−1^	0.68 mg g^−1^	—	168
Alginate–clay shell and magnetite–gelatin core composite	Heavy metal ion	Cd^2+^	Langmuir	PSO	pH = 7, using adsorbent concentration = 500 mg, and *t* = 30 min	43.86 mg g^−1^	The adsorption efficiency was 98.0% ±0.9%	169
CH@AL composite beads	Heavy metal ion	Cu^2+^	Langmuir	PSO	pH = 3.2 adsorbent dosage = 20 mg, and *t* = 190 min	92.44 mg g^−1^	—	170
2 : 1-Mt/SA composite beads	Antibiotic	TC	Langmuir	PSO	pH = 5, adsorbent dosage = 0.10 g, and *T* = 328 K	745 mg g^−1^	—	166
MT/SA/OD (oven-dried)	Antibiotic	TC	Langmuir	PSO	pH = 5 ant *t* = 4 h	499 mg g^−1^	—	171
CA-Al-KABs microspheres	Antibiotic	CIP	Langmuir	PSO	pH = 4, adsorbent dosage = 0.04 mg/50 mL, and *T* = 308.15 K	68.36 mg g^−1^		172
SCGG	Dye	MB	Freundlich	PSO	pH = 7, adsorbent dosage = 1.0 g, *T* = 35 °C, MB concentration 50 mg L^−1^, and *t* = 24 h	133.24 mg g^−1^	The adsorption rate 82% after five cycles	173
NaAlg/BLAC/OMMT	Dyes	Eriochrome black (EBT) and MB	Freundlich	EBT (PSO) and MB (PFO)	pH = 7, adsorbent dosage = 10 mg, and *t* = 24 h	136.98 mg g^−1^ for EBT and 66.66 mg g^−1^ for MB	—	174
Biochar-incorporated SA- kaolin beads	Pesticide	Naphthalene	Langmuir	PSO	Adsorbent dose = 20 g L^−1^, *t* = 90 min, and pH = 4	—	The adsorption rate 81.237% after five cycles	175
*N*-Bent-NFe_3_O_4_-Sod.Alg	Pesticide	Chlorpyrifos (CPS)	Langmuir	PSO	pH = 7.0 initial concentration of CPS = 20.0 mg L^−1^, *T* = 20 °C, and *t* = 80.0 min	29.17 mg g^−1^	—	176

### 4.5. Sodium alginate–other biomolecule composites

The selection of cost-effective, biocompatible, and biodegradable biopolymer gels as adsorbents represents a modern adsorption strategy aimed at mitigating secondary environmental contamination.^177,178^ The polymer's upper surface contains numerous functional groups (–OH, –COOH, –NH_2_, *etc.*) that provide additional active sites for pollutant adsorption or for altering other target functional groups. Table 5 highlights the adsorption performance of various sodium alginate–other biomolecule composites.

**Table 5:** Adsorption of water pollutants using SA–other biomolecule composites

Adsorbent	Type of pollutants	Pollutant	Isotherm model	Kinetic model	Adsorption conditions	Adsorption capacity	Reusability	Ref.
Ts-Car/Alg	Heavy metal ion	Pb^2+^	Freundlich	PSO	pH = 5.3, adsorbent dosage = 0.15 g, Pb^2+^ concentration = 100 mL, and *t* = 120 min	74 mg g^−1^	The removal rate 80% after the second cycle	225
CTS/SA/Ca^2+^ PCDNH	Heavy metal ions	Cd^2+^, Cu^2+^, and Pb^2+^	Langmuir–Freundlich	Both PFO and PSO	*T* = 298 K, adsorbent dosage = 25 mg, and *t* = 8 h	99.46 mg g^−1^ for Cd^2+^, 70.32 mg g^−1^ for Cu^2+^, and 278.30 mg g^−1^ for Pb^2+^	—	226
CSM/SA	Heavy metal ions	Cr(vi) and Pb^2+^	Langmuir	PFO for Pb^2+^ and PSO for Cr(vi)	pH = 3.0 for Cr(vi) and 5.2 for Pb^2+^, *T* = 25 °C, adsorbent dosage = 0.06 g, and *t* = 250	16 mg g^−1^ for Cr(vi) and180 mg g^−1^ for Pb^2+^	—	53
MCPS	Heavy metal ions	Cu^2+^ and Cr(vi)	Langmuir and Koble–Corrigan models		pH = 3 for Cr(vi) and pH = 6 for Cu^2+^	87.53 mg g^−1^ for Cr(vi) and 351.03 mg g^−1^ for Cu^2+^	—	227
CS-LDHs-SA	Heavy metal ions	Cu^2+^ and Pb^2+^	Langmuir and Koble–Corrigan models	PFO and PSO for Cu^2+^ and PFO for Pb^2+^	*T* = 303 K, adsorbent dosage = 10.2 mg, and pH 5.3	106 mg g^−1^ for Cu^2+^ and 155 mg/for Pb^2+^	—	183
CS/SA-FeMT composite	Phosphate	Phosphate	Langmuir	PSO	After five cycles, the adsorption capacity decreased from 55.90 mg g^−1^ to 45.64 mg g^−1^, a decrease of 18.35%	88.3 mg g^−1^	The adsorption rate was decrease 18.35% after five cycles	228
FSSEP	Antibiotic	CIP	Langmuir	PSO	pH = 5	423.69 mg g^−1^	After five cycles, the removal efficiency was 78.31%	229
DCNC	Antibiotic	Doxycycline (DOXY)	Freundlich	PSO	pH = 7, DOXY solution = 5 mL, *T* = 45 °C, and adsorbent dosage = 0.1 g	594.6 mg g^−1^	After five cycles, the removal efficiency remained after 80%	224
Chitosan/sodium alginate composite	Dye	MB	Langmuir	PSO	Adsorbent dosage = 10 mg, MB concentration = 500 mg L^−1^, pH = 5.81 for MB	1488.1 mg g^−1^ for MB	The adsorption capacity for MB was 697.5 mg g^−1^ after four regeneration cycles	230
AlCu-LDH/CMC-Alg hydrogel beads	Pesticides	Diquat (DQ)	Langmuir	PSO	pH = 8 and adsorbent dosage = 0.02	302.6 mg g^−1^	The adsorption rate 88.2% after six cycles	231

#### 4.5.1. Sodium alginate–chitosan composites

**Fig. 11:**
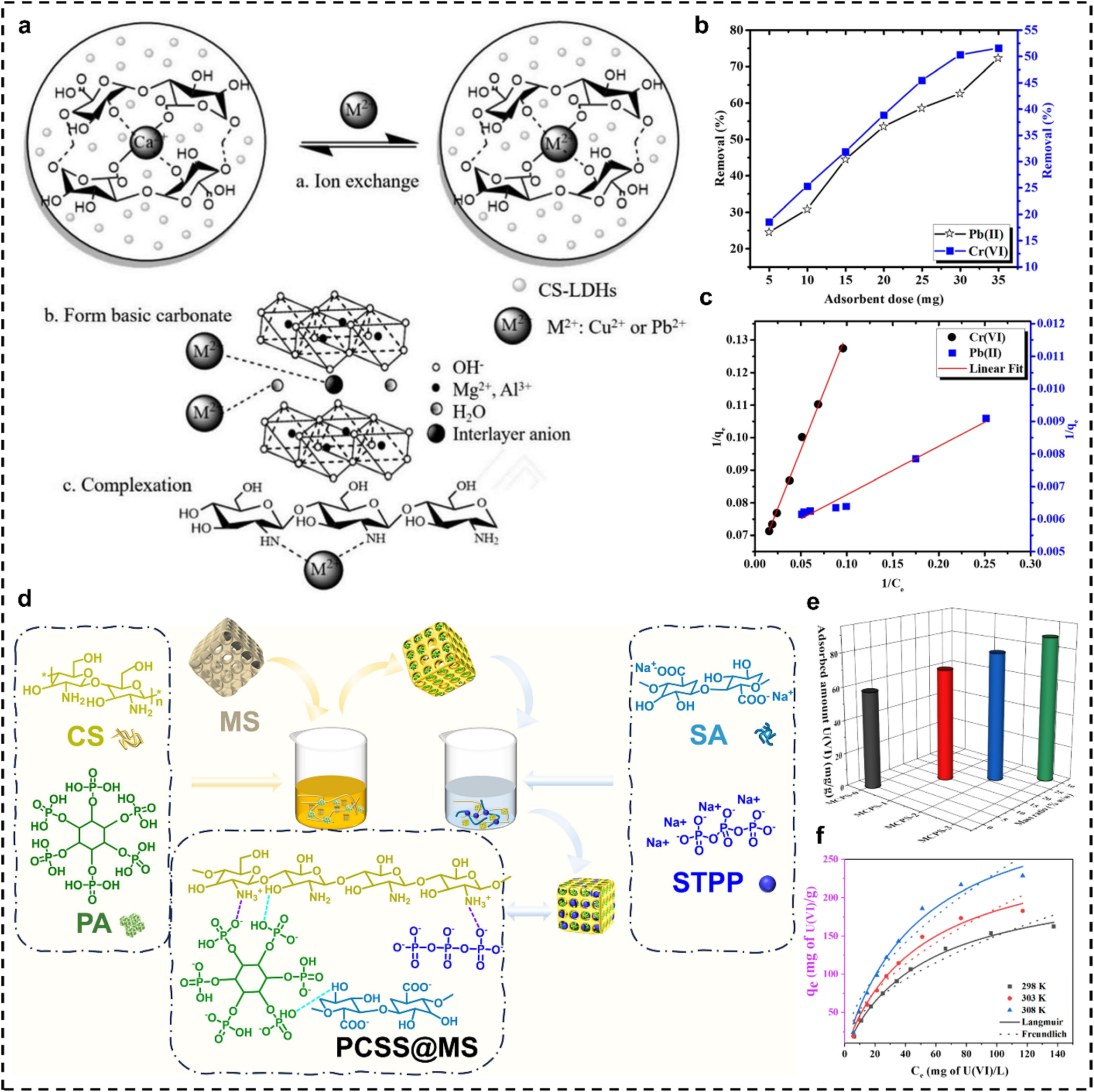
(a) Proposed removal mechanisms of Cu^2+^ and Pb^2+^ using CS-LDHs-SA,^183^ (b) influence of adsorbent dose on adsorption of Cr(vi) and Pb^2+^, (c) Langmuir isotherm plot for Cr(vi) and Pb^2+^ adsorption using CSM/SA composite beads,^53^ (d) illustration for the synthesis process of PCSS@MS,^184^ (e) the comparative adsorption performance of various MCPS with various WO_3_ amounts, and (f) isothermal plots of adsorption of U(vi) using MCPS-3 composite.^185^

Chitosan (CS), a natural biopolymer, is commonly found in the shells of crustaceans and other animals.^179^ CS is widely employed in the environmental and chemical fields due to its high adsorption capacity, bacteriostatic properties, biodegradability, and low toxicity.^180,181^ CS is composed of amino and hydroxyl groups, which provide additional binding sites for pollutants. But its properties, including a small specific surface area, low thermal stability, weak mechanical strength, and low acid stability, limit the practical application of CS. Thus, its chemical modification can address these challenges and enable the synthesis of composites with superior adsorption performance.^182^ CS is often integrated with SA to form composites for the removal of pesticides, heavy metal ions, dyes, and phosphate. For example, CS-LDHs-SA hydrogel beads were prepared as an effective adsorbent for Cu^2+^ and Pb^2+^ adsorption.^183^ SA developed as a robust hydrogel capable of ion exchange; CS supplied many amino groups for metal coordination; and layered double hydroxides (LDHs) provided positively charged architectures with an affinity for metal ions. The incorporation of these materials resulted in an organic–inorganic composite with several advantages, including improved adsorption performance, enhanced structural stability, and abundant functional binding sites. Thus, the CS-LDHs-SA composite hydrogel exhibited *q*_max_ values of 155 and 106 mg g^−1^ for Pb^2+^ and Cu^2+^, respectively. The addition of PEG improved internal porosity, whereas the inclusion of CS and SA yielded a composite with dual functional sites for ion exchange and metal ion complexation (Fig. 11a). The combined design elements provide a logical strategy for overcoming several intrinsic constraints of traditional alginate-based adsorbents. Ablouh *et al.*^53^ prepared a green adsorbent using SA hybrid beads and CS microspheres (CSM/SA) to adsorb Pb^2+^ and Cr(vi) from aqueous solutions. The removal efficiencies reached 51% for Cr(vi) and 72% for Pb^2+^ at an adsorbent dosage of 35 mg (Fig. 11b). The CSM/SA composite achieved *q*_max_ values of 16 mg g^−1^ for Cr(vi) and 180 mg g^−1^ for Pb^2+^ (Fig. 11c). This study indicates that CSM/SA hybrid beads could provide a sustainable approach to the highly effective adsorption of heavy metal ions. Similarly, a new PCSS@MS composite was prepared by cross-linking phytic acid-modified CS, SA, and melamine sponge (Fig. 11d).^184^ The resultant PCSS@MS composite was used to assess U(vi) adsorption performance. XRD and SEM analyses indicated that the 3D porous structure was stable. The experimental results indicated that the PCSS@MS composite exhibited superior U(vi) adsorption capacity over a wide pH range, with a *q*_max_ of 297.86 mg g^−1^. Additionally, the PCSS@MS composite retained 70.8% U(vi) adsorption efficiency after four cycles. XPS and FTIR studies indicated that phosphate groups played the primary role in uranium adsorption, with additional involvement of hydroxyl, carboxyl, and amino groups. In another study, ethyl acetoacetate was attached to the chitosan *via* a polar mechanism. The primary goal of this functionalization was to introduce the 3-dioxo moiety on the chitosan backbone.^185^ Then, functionalized CS (EAA-CS) was intercalated into a phosphate-containing alginate network (PASA). Subsequently, WO_3_-doped composites with various WO_3_-to-PASA mass ratios were prepared to adsorb uranium. Among the synthesized composites, the MCPS-3 composite beads with a 30.0% w/w mass ratio exhibited superior uranium adsorption capacity (Fig. 11e). The appearance of two U4f peaks at 381.36 and 392.25 eV confirmed successful uranium adsorption. The adsorption of U(vi) obeyed the PSO (*q*_e,th_ ≈ 116.88 mg g^−1^ and *R*^2^ = 0.99) kinetic model, and equilibrium sorption data conformed with Temkin (*R*^2^ = 0.99) and Langmuir (*q*_m_ = 343.85 mg g^−1^ and *R*^2^ = 0.99) (Fig. 11f) isotherm models. The thermodynamic study indicated that the removal of U(vi) onto the MCPS-3 was endothermic, spontaneous, and feasible. These results suggest that MCPS-3 may be a superior hydrogel-based biomaterial for uranium recovery.

**Fig. 12:**
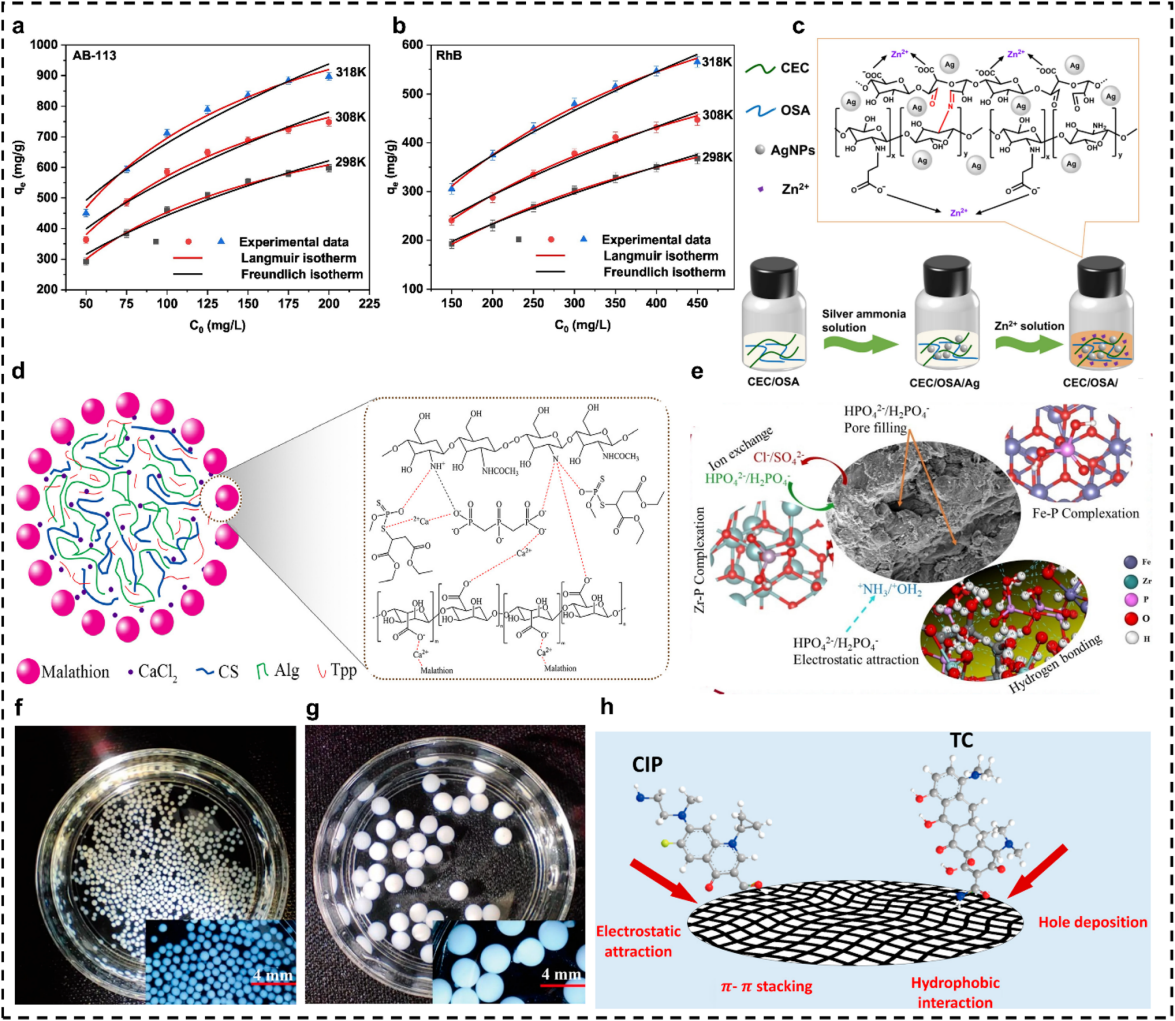
Isothermal plots of the adsorption of (a) AB-113 and (b) RhB using CESA composite sponges.^186^ (c) Illustration of the preparation process for CEC/OSA/Ag1/Zn^3^ hydrogel,^187^ (d) representation of proposed interactions of malathion with the chitosan-alginate,^191^ and (e) possible adsorption mechanism of PO_4_^3−^ using Zr/Fe/CS/Alg composite.^192^ The snapshot of (f) SC-400 and (g) SC-2000 microbeads, and (h) representation of the removal mechanism of TC and CIP using SA/CMCS microbeads.^193^

The coexistence of cationic and anionic dyes in wastewater demands the development of a promising adsorbent for their simultaneous removal. To address this issue, Zhao and his colleagues prepared an amphoteric composite sponge by integrating CS with electrospun SA nanofiber employing lyophilization.^186^ The resultant composite exhibited a remarkable microstructure characterized by nanoscale fibers and interconnected pores, along with numerous functional groups that are advantageous for adsorption. The as-prepared adsorbent exhibited adsorption capacities of 926.2 and 695.4 mg g^−1^ for Acid Blue-113 (AB-113) and Rhodamine B (RhB), respectively (Fig. 12a and b). Notably, it exhibited exceptional adsorption capacity for a binary solution system comprising cationic and anionic dyes. Lai *et al.*^187^ synthesized a novel hydrogel by crosslinking oxidized sodium alginate (OSA) and carboxyethyl chitosan (CEC) with the introduction of Zn^2+^ and silver nanoparticles (Fig. 12c). The OSA served dual roles as reducing and cross-linking agents, promoting the quick reduction of Ag ions *via* the Ag mirror reaction, thereby forming AgNPs. The gel network became more densely organized upon the addition of silver nanoparticles, increasing the gel's hardness. Additionally, the integration of Zn^2+^ further enhanced the hydrogel's characteristics. The adsorption experiments revealed that the CEC/OSA/Ag^1^/Zn^3^ hydrogel exhibited *q*_max_ values of 79.15 and 157.23 mg g^−1^ for MB and Congo red (CR), respectively. Based on the kinetic study, CR and MB adsorption obeyed the PSO model. According to the isotherm study, CR removal conforms to the Langmuir model, whereas MB adsorption aligns with the Freundlich model. This research provides a sustainable and effective strategy for addressing dye wastewater treatment issues. Mokeddem *et al.*^188^ studied the adsorption capacity of Coralene Dark Red2B (DR) and CR onto CS and SA biopolymer films. Using a “solvent-cast” technique, the two biopolymers were incorporated at various mass ratios to introduce their distinct qualities and assess their potential to adsorb the dyes. The kinetic study indicated that the Alg/Cs biofilms at different proportions reached equilibrium within 30–180 min, with outstanding adsorption capacities ranging from 222.30 to 842.36 mg g^−1^. The characterization results indicated that hydrogen bonding and electrostatic interactions were primarily responsible for the adsorption of DR and CR onto the biopolymer film.

Due to the variety of plant pests and the overuse and unregulated use of these substances, agricultural effluents contain extremely harmful pesticides. The pollutants released from agricultural pesticides are carcinogenic and nondegradable.^189,190^ The presence of pesticides in water harms both human health and the aquatic ecosystem over time. Thus, an easily accessible and cost-effective chitosan-alginate composite was synthesized *via* a microemulsion approach to adsorb malathion from agricultural wastewater (Fig. 12d).^191^ The highest elimination rate was 82.35% with an initial dosage of 10 mg L^−1^, an adsorption time of 20 min, and an adsorbent concentration of 0.18 g. To evaluate the model's importance and adequacy, an analysis of variance (ANOVA) was used. The results showed that the quadratic model was optimal for predicting malathion removal. The PSO and Langmuir models were suitable for the kinetic and isotherm analyses, respectively. The outcomes indicated that the CS-SA biopolymer was an affordable and effective adsorbent for removing malathion from agricultural wastewater. Ahmed *et al.* prepared a new Zr/Fe/CS/Alg using an *in situ* reduction approach for phosphate adsorption (Fig. 12e).^192^ The Zr/Fe/CS/Alg achieved a *q*_max_ of 221.72 mg g^−1^ for PO_4_^3−^. The adsorption mechanism was shown through XPS, FTIR, and zeta potential measurements. The higher pH_ZPC_ (6.7) of Zr/Fe/CS/Alg hydrogel beads indicates that HPO_4_^2−^/H_2_PO_4_^−^ can be captured *via* electrostatic attraction at pH < 7, as the surface becomes protonated below pH_PZC_. Therefore, protonating the surface creates ^+^NH_3_ and ^+^OH_2_ sites that attract HPO_4_^2−^/H_2_PO_4_^−^*via* electrostatic interactions. After phosphate adsorption, new FTIR peaks appeared and exhibited a spectral shift. For example, the intensities of the COO^−^ and NH_2_ groups decreased, indicating that these groups were involved in phosphate adsorption. Similarly, two new peaks were identified at 487 and 447 cm^−1^, confirming M–O–P vibrations (M = Zr/Fe) on the surface and suggesting phosphate adsorption. Furthermore, all peak intensities on the Zr/Fe/CS/Alg were markedly reduced after phosphate uptake, indicating interaction between HPO_4_^2−^/H_2_PO_4_^−^ and the adsorbent. The deconvoluted XPS peaks show a slight shift of Zr 3d peaks after phosphate uptake, indicating electron transfer between HPO_4_^2−^/H_2_PO_4_^−^ and Zr, resulting in Zr–O–P inner–sphere complex formation. Likewise, the downward shift of the Fe 2p peaks indicates the formation of complexes between HPO_4_^2−^/H_2_PO_4_^−^ and Fe. Meanwhile, the P 2p peak was observed at 131.83 eV, a lower binding energy than that of H_2_PO_4_^−^, suggesting phosphate interacting with the surface. The potential for complexation *via* electron transfer was demonstrated by the Monte Carlo (MC) and Density Functional Theory (DFT) calculations. According to experimental and theoretical data, potential adsorption mechanisms involved pore-filling processes, ion exchange, electrostatic interactions, inner-sphere complexation, and hydrogen bonding. Qian *et al.*^193^ efficiently prepared SA/CMCS hydrogel microbeads using the electrostatic spray method to enhance the adsorption of CIP and TC. Hydrogels with microbead sizes of ∼400 µm (SC-400) (Fig. 12f) and ∼2000 µm (SC-2000) (Fig. 12g) were selected for CIP and TC removal in both binary and single systems. The resulting hydrogel microbeads demonstrated enhanced adsorption efficacy, with maximum adsorption at 25 °C and pH 7. The experimental results indicated that both binary and single systems were better fit by the Temkin and Freundlich models, demonstrating their appropriateness for elucidating the adsorption mechanisms. These adsorption mechanisms primarily involve electrostatic interactions between adsorbents and CIP/TC antibiotics (Fig. 12h).

#### 4.5.2. Sodium alginate–polyethyleneimine composites

**Fig. 13:**
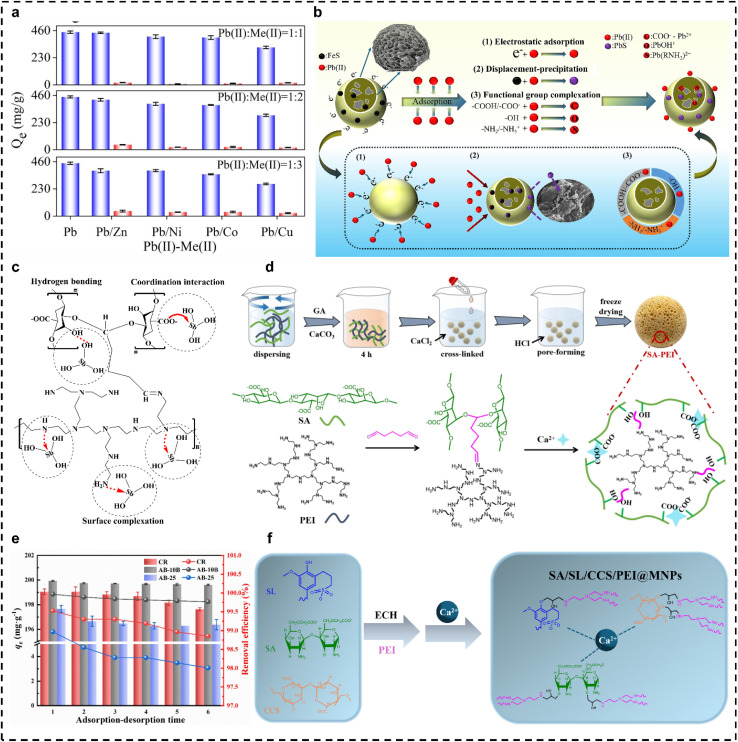
(a) Binary coexisting ion systems, (b) illustration of the removal mechanism of Pb^2+^ using the FeS/PEI/Alg,^195^ (c) representation of the removal mechanism of Sb by PEI/ALG,^201^ (d) illustration of the preparation process of SA-PEI aerogel,^202^ (e) adsorption/desorption cycles of AB-25, AB-10B, and CR using SA/PEI/PEG,^207^ and (f) illustration of the synthesis route of SA/SL/CCS/PEI@MNPs.^208^

Polyethyleneimine (PEI) is a type of water-soluble polyamine. Various amine groups are present along its molecular chain, including –NH_2_, –NH^−^, and =N^−^, indicating a remarkable affinity for metal ions and extensive use in adsorbent modification.^194^ The incorporation of PEI improves the 3D network architecture of SA and introduces amino groups (–NH_2_,– NH, and =N–). The PEI/SA composite comprises various groups, including imine (=N–), imine (–NH^−^), amino (–NH_2_), carboxyl (–COOH), and hydroxyl (–OH). Motivated by this, (FeS/PEI/Alg) gel was developed through the modification of alginate by ferrous nanosulfide (FeS) and PEI for the removal of lead.^195^ The characterization consequences indicated that the FeS/PEI/Alg composite effectively adsorbed lead ions *via* displacement-precipitation (FeS) pathways, complexation (NH_2_, OH, and COOH), and electrostatic attraction. Batch studies indicated that the FeS/PEI/Alg composite exhibited superior, rapid adsorption performance, achieving an removal capacity of 565.0 mg g^−1^. Moreover, FeS/PEI/Alg demonstrated remarkable anti-interference characteristics in binary (Fig. 13a) and five-component systems (Zn^2+^, Ni^2+^, Co^2+^, Cu^2+^, and Pb^2+^). Characterization results indicated that electrostatic attraction, functional group complexation, and substitution and precipitation of inorganic components primarily contributed to Pb^2+^ adsorption, as illustrated in Fig. 13b. In another study, a highly efficient and natural ALG/PEI hydrogel was prepared for the removal of Cu^2+^ and Pb^2+^.^196^ The ALG/PEI hydrogel achieved maximum Cu^2+^ and Pb^2+^ adsorption capacities of 344.8 and 322.6 mg g^−1^, which were ∼1.6 and ∼1.5 times superior to the adsorption capacities of pure ALG. The adsorption of Pb^2+^ and Cu^2+^ ions by ALG/PEI composite hydrogel obeyed the PSO and Langmuir models. Similarly, an Alg-PEI hybrid aerogel was synthesized *via* a straightforward single-step method to uptake Cu^2+^.^197^ The resultant Alg-PEI hybrid aerogel demonstrated superior affinity for Cu^2+^ and adsorbed 95.1% of the Cu^2+^ from a 0.1 mM Cu^2+^ solution. In the presence of Cd^2+^, Co^2+^, Fe^2+^, and Cr^3+^, the Alg-PEI selectivity coefficients for Cu^2+^ were greater than 10. Alg-PEI hybrid aerogel achieved the highest Cu^2+^ uptake capacity of 214.4 mg g^−1^. Studies of the removal mechanism indicated that the Alg-PEI hybrid adsorbent was bound to Cu^2+^*via* chemical coordination and ion exchange. Moreover, Sb is widely used in lead-acid batteries, flame retardants, military equipment, and other applications. Antimony (Sb) is a hazardous element to humans and is categorized as highly carcinogenic by the World Health Organization (WHO).^198^ Compared to Sb(v), Sb(iii) is tenfold more hazardous.^199^ Wastewater released during the smelting and mining of Sb ore can contain Sb concentrations ranging from 100 to 7000 mg L^−1^.^200^ Because Sb is difficult to degrade in the natural environment, it tends to accumulate continuously and can cause serious environmental contamination. Yang *et al.*^201^ successfully modified SA with PEI (PEI/ALG) for the first time, enabling the removal of Sb(iii). The PEI/ALG composite exhibited an excellent maximum Sb(iii) removal capacity of 743 mg g^−1^ and retained a 90–98% removal rate after eight cycles. DFT computations indicated that the –N=, –NH–, –NH_2_, –COOH, and OH functional groups interacted with Sb. Additionally, experimental results indicated that the mechanism of PEI/ALG composite adsorption of antimony was primarily involved coordination between –COOH, –OH, and Sb(iii) and hydrogen bonding (Fig. 13c). Zhao *et al.*^202^ synthesized a SA–PEI composite aerogel using SA and PEI for the removal of Cu^2+^ and phytic acid (PA). SA-PEI successfully adsorbed PA *via* electrostatic interactions and hydrogen bonding, with a *q*_max_ of 269.39 mg g^−1^. Then, phytic acid-adsorbed SA-PEI (SA-PEI@PA) was recovered and reused for Cu^2+^ adsorption. The SA-PEI@PA adsorbent achieved a higher *q*_max_ (188.74 mg g^−1^) and a higher removal rate than the single adsorption system for Cu^2+^. Thermodynamic, isotherm, and kinetics studies indicated that Cu^2+^ adsorption obeyed an endothermic, spontaneous, and entropy-driven single-layer chemical adsorption process. The experimental findings revealed that the removal mechanism mainly involved coordination bonding *via* phosphate and amino groups to form Cu–N or Cu–O bonds, and electrostatic attraction between negatively charged phosphate/carboxyl groups and Cu^2+^. Huang *et al.*^203^ prepared reusable and cost-effective SA/PEI by a simple crosslinking method (Fig. 13d). Crosslinking PEI with SA improved its mechanical strength and thermal stability. Thermodynamic parameters revealed that the uptake of U(vi) onto the SA/PEI adsorbent was spontaneous and endothermic. The U(vi) adsorption was fitted to the Langmuir and PSO models, indicating that the adsorption mechanism was monolayer chemosorption. The SA/PEI composite exhibited a *q*_max_ of 353.09 mg g^−1^ for U(vi).

Similarly, indigo derivatives^204^ (including acid blue-25 (AB-25), amino back 10B (AB-10B), Congo red (CR), and Indigo), azo compounds,^205^ and anthraquinones^204^ are released in large amounts, resulting in degradation of terrestrial ecology. Additionally, when interacting with the human body, these toxic dyes can cause respiratory and skin diseases.^206^ To remove these toxic dyes, a SA/PEI/PEG composite was prepared using polyethylene glycol diglycidyl ether (PEG), SA, and PEI through one-pot gelation of the SA/PEI/PEG solution.^207^ The SA/PEI/PEG composite exhibited remarkable adsorption capacities for CR (2782 mg g^−1^), AB-25 (4222 mg g^−1^), and AB-10B (1369). Additionally, the SA/PEI/PEG composite exhibited outstanding reusability, maintaining a 98% removal rate for three anionic dyes over six adsorption–desorption cycles (Fig. 13e). Moreover, the interfering cations, including Mg^2+^, Ca^2+^, K^+^, and Na^+^, facilitated the adsorption of CR, AB-25, and AB-10B dyes. The EDS, SEM, XPS, and FTIR analyses confirmed that hydrogen bonding and electrostatic interaction between the dyes and the SA/PEI/PEG composite were primary drivers of dye adsorption. Wang *et al.*^208^ prepared a SA/SL/CCS/PEI@MNPs composite through a single-pot approach using SA, carboxylated chitosan (CCS), PEI, sodium lignosulfonate (SL), and magnetic nanoparticles (MNPs), as depicted in Fig. 13f. The outcome revealed that the integration of PEI, CCS, and SL provided abundant imine/amino, carboxylate, and sulfonate groups, thereby improving dye adsorption performance. In an acidic environment, the as-prepared adsorbent exhibited adsorption capacities exceeding 550 mg g^−1^ for indigo carmine, reactive black-5, and tartrazine dyes. In contrast, adsorption capacities for neutral red, methyl violet, and methylene blue exceeded 1900 mg g^−1^ under an alkaline environment. Characterization results indicated that the dye removal onto the adsorbent was primarily due to π–π interaction and electrostatic attraction.

#### 4.5.3. Sodium alginate–cellulose composites

**Fig. 14:**
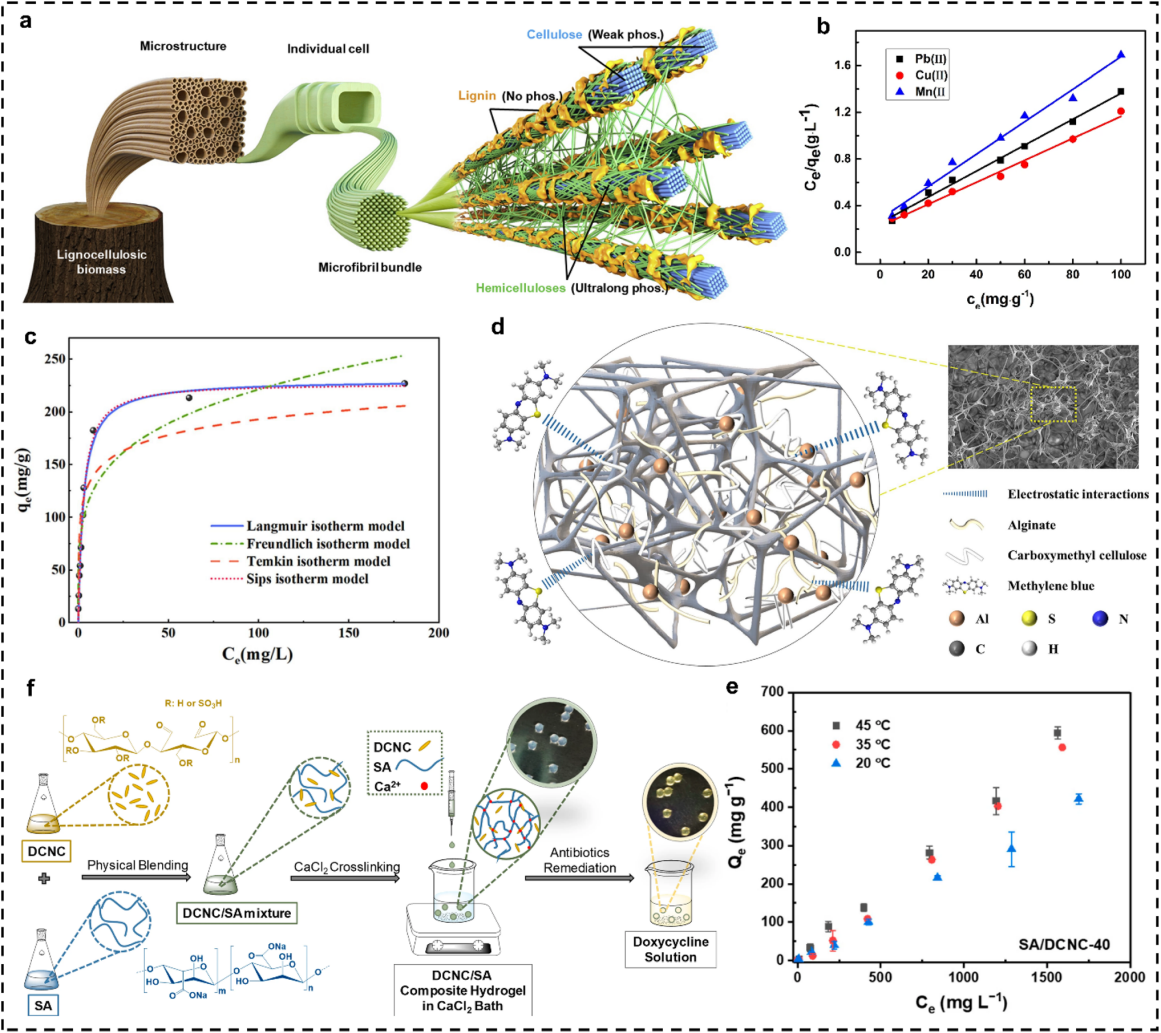
(a) Illustration of the constituents of lignocellulosic biomass and their associated phosphorescent properties,^216^ (b) Langmuir isotherm plot for Mn^2+^, Pb^2+^, and Cu^2+^ ions adsorptions,^218^ (c) isothermal plots of the adsorption of MB using SA/CMC-MeS, (d) illustration of the removal mechanism of MB onto the SA/CMC-MeS,^222^ (e) synthesis of SA-DCNC hydrogel beads, and (f) adsorption isotherm of DOXY at different temperatures.^224^

Cellulose is a naturally occurring substance found in plants and other sources, and it is the most abundant and sustainable natural polymer.^209,210^ Cellulose is composed of β-d-glucose/β-d-glucopyranose units connected by β-1,4-glycosidic bonds.^211–213^ It can be separated from several sources, including bacteria, algae, wood, and plants.^214,215^Fig. 14a shows the laminar architecture at the nanomolecular and micromolecular levels.^216^ It is recognized as an outstanding carrier for composite materials due to its sustainability, natural abundance, biocompatibility, and good biodegradability. Significantly, it's a promising class of adsorbent substances.^217^ Moreover, cellulose is a perfect blend modifier for SA. For instance, a new composite hydrogel was efficiently prepared using carboxymethyl cellulose (CMC) and SA as the backbone, and it was *in situ* filled with Fe_3_O_4_ nanoparticles for the removal of Cu^2+^, Mn^2+^, and Pb^2+^.^218^ The resultant composite hydrogel exhibited maximum adsorption capacities of 105.93, 89.49, and 71.83 for Cu^2+^, Mn^2+^, and Pb^2+^, respectively (Fig. 14b). In another study, SA-CMC gel beads were developed by cross-linking and blending CMC and SA in FeCl_3_ and CaCl_2_ solutions. The gel beads exhibited a limited adsorption capacity for Pb^2+^. Due to its low porosity and high internal structural density, it exhibited excellent treatment performance for low-dosage wastewater. It is challenging to fulfill the demand for practical applications. For practical applications, Chen *et al.*^219^ prepared the SA/CNF hydrogel by integrating SA with CNF. The resultant hydrogel microspheres effectively remove Cd^2+^, Pb^2+^, and Cu^2+^. The adsorption of Cd^2+^, Pb^2+^, and Cu^2+^ obeyed the PSO kinetic model and the Langmuir equation, revealing that the uptake of Cd^2+^, Pb^2+^, and Cu^2+^ was primarily chemosorption. The SA/CNF hydrogel microspheres achieved highest removal capacity of 544.66 mg g^−1^ for Pb^2+^ at 20 °C. Nasr *et al.*^220^ proposed a novel recyclable SA/cell./GO adsorbent composed of GO, SA, and cellulose. The experimental outcomes revealed that the removal rates of Pb^2+^ and Cu^2+^ were 99.5% and 100%, respectively, after 40 and 60 min. The adsorption processes were more consistent with the PSO kinetic model and the Freundlich model.

Novel cellulose-Na-Alg adsorbent was synthesized for the adsorption of MB molecules.^221^ To synthesize a biocomposite with Na-Alg and clay, cellulose was derived from a paper mill waste. The outcome indicated that the adsorption data were consistent with the PSO model. The thermodynamic study indicated that the removal of MB dye onto the cellulose-Na-Alg adsorbent was spontaneous, exothermic, and feasible. Zeng *et al.*^222^ synthesized a melamine (MeS) sponge-based adsorbent by a facile crosslinking approach using MeS, CMC, and SA for the adsorption of MB dye. The outcomes indicated that the PSO and Langmuir models better described the removal process. The SA/CMC-MeS composite achieved a theoretical removal capacity of 230 mg g^−1^ (Fig. 14c). The FTIR and pH studies confirmed that the removal of the dye onto the adsorbent was primarily due to the electrostatic attraction between cationic dye (S)^+^ in solution and anionic COO^−^ functional groups of the adsorbent (Fig. 14d). Fu *et al.*^223^ modified the CMC/SA hydrogel beads with bentonite (Be) and CaCO_3_. The homogeneous surface confirmed the compatibility between Be and CaCO_3_, as evidenced by SEM images. The as-prepared beads achieved the maximum removal capacities of 90.31 mg g^−1^ for phosphate and 142.15 mg g^−1^ for MB dye, respectively. In another study, a novel SA-DCNC hydrogel bead was synthesized using aldehyde cellulose nanocrystals (DCNC) and SA *via* a cross-linking process for the adsorption of doxycycline (DOXY) and three other TCs (Fig. 14e).^224^ The resultant SA-DCNC hydrogel achieved removal performances of 649.9 and 421.5 mg g^−1^ for DOXY at pH 11 and pH 7, respectively. Additionally, the adsorption capacity of the SA-DCNC hydrogel increased with temperature, as shown in Fig. 14f. The XPS study confirmed that the removal of DOXY by sodium alginate-DCNC beads involved synergistic effects of surface deposition/pore diffusion, hydrogen bonding, electrostatic interactions, and chemical reactions.

## 5. Pollutant speciation

The effectiveness of pollutant adsorption on SA adsorbents depends heavily on pollutant speciation, which reflects their charge state and interactions with the adsorbent surface. Pollutant speciation is primarily influenced by solution pH, dissolved organic matter, competing ions, ionic strength, and redox potential. Adsorption of contaminants mainly occurs *via* interactions between the surface functional groups of adsorbents and pollutant species. Consequently, pollutant speciation markedly affects adsorption capacity, mechanisms, kinetics, and selectivity. Heavy metal ions, including Ni^2+^, Cu^2+^, Cd^2+^, and Pb^2+^, exhibit significant pH-dependent speciation, which markedly affects their binding to the sodium alginate-based adsorbent. Metal ions primarily occur as hydrated free cations, such as Ni^2+^, Cu^2+^, Cd^2+^, and Pb^2+^, under acidic conditions. At higher pH levels, hydrolysis yields species such as Cd(OH)^+^, Cu(OH)^+^, and Pb(OH)^+^.^167,225,232^ However, the adsorption of heavy metal ions onto the SA depends on the point of zero charge (pH_PZC_). The pH_pzc_ values of the SA-based adsorbent varied with the type of material attached to SA to form the SA composite. For instance, the pHpzc of the SA/CMC/SKL hydrogel was approximately 2.5.^161^ Thus, the SA/CMC/SKL hydrogel carries a positive charge at pH below 2.5 and a negative charge at pH above 2.5. At pH < 3.0, the abundant H^+^ ions in the solution and the metal cation experience substantial electrostatic repulsion, resulting in minimal adsorption. At pH values greater than 3, the negatively charged hydrogel readily attaches to heavy metals *via* electrostatic attraction. Similarly, a novel Fe/Mg–SA adsorbent was prepared using Mg^2+^ and Fe^3+^ as cross-linkers for As(v) adsorption.^233^ The Fe/Mg–SA exhibited higher adsorption capacity under acidic conditions than under alkaline conditions, reaching a maximum of 37.0 mg g^−1^. The pH_pzc_ value of Fe/Mg–SA composites was 7.0, indicating that the surface becomes positively charged below pH_pzc_. The adsorption of the arsenate anion was enhanced by electrostatic interactions. However, at very low pH, Fe^3+^ tends to dissolve, which negatively affects As(v) adsorption. When pH exceeds 7.0, the Fe/Mg–SA surface carries a negative charge due to deprotonation, thereby weakening electrostatic interactions and the chelation of arsenate ions with the adsorption surface. This reduction impairs the efficiency of As(v) removal, thereby decreasing adsorption capacity.

Similarly, phosphate exists in various forms, such as PO_4_^3−^, HPO_4_^2−^, H_2_PO_4_^−^, and H_3_PO_4_, depending on pH in aquatic environments. Thus, the pH of the initial solution influences the phosphate adsorption performance. Thus, the pH of the initial solution influences phosphate adsorption performance, while the adsorbent's surface charge and adsorption capacity are also affected. For example, MXene-embedded Zr-crosslinked SA (MX-ZrSA) beads were prepared to remove phosphate under various conditions.^125^ MX-ZrSA exhibited a maximum phosphate adsorption capacity of 135.75 mg g^−1^ at pH 4.12. The zeta potential of MX–ZrSA composite beads gradually shifted from positive to negative, with the zero-charge point at pH 3.3. At a pH of 2–3.3, the surface of the MX–ZrSA beads was positively charged, and within this pH range, the negatively charged H_3_PO_4_ and H_2_PO_4_^−^ species were the predominant phosphate forms in the solution. Thus, in this pH range, phosphate adsorption occurred *via* electrostatic interactions. When the pH rose above 3.3, the zeta potential of the MX–ZrSA beads composite decreased significantly, leading to a reduction in adsorption capacity due to electrostatic repulsion and competition from OH^−^ ions.

Organic pollutants, such as pesticides, antibiotics, and dyes, contain various ionizable functional groups, leading to pH-dependent speciation. For example, Tetracycline exists as a cationic form at pH values below 3.3 due to the dimethylammonium group. When the pH is between 3.3 and 7.7, it appears as a zwitterionic species due to deprotonation of the anionic form. TC take on an anionic form when pH exceeds 7.7 because the tri-carbonyl system undergoes deprotonation.^234,235^ Similarly, CIP has two p*K*_a_ values (p*K*_a1_ = 5.68 and p*K*_a2_ = 8.66) corresponding to the –COOH and –NH functional groups, respectively. CIP in aqueous solution occurs in three forms: as an anion, an amphoteric ion, and a cation, depending on pH. CIP exists as a cation (CIP^+^) below pH 5.9 and as an anion (CIP^−^) above pH 8.9.^236,237^ Consequently, adsorption primarily occurs *via* surface complexation, π–π interactions, hydrophobic interactions, and hydrogen bonding. Similar behaviors have been observed across many emerging pollutants and pharmaceutical residues, emphasizing the importance of accounting for pollutant speciation when designing adsorbents and optimizing processes.

## 6. Adsorption mechanisms

It is essential to understand the mechanisms by which pollutants are removed from wastewater onto sodium alginate-based composites to develop a high-performance adsorbent for wastewater treatment. SA biopolymer is composed of –COO– and –OH functional groups, providing several adsorption sites that facilitate the removal of pesticides, radioactive metal ions, heavy metal ions, dyes, and phosphate *via* hydrogen bonding, electrostatic forces, complexation, and Ion exchange.

### 6.1. Type of interactions

The adsorption of water pollutants involves various interactions, including hydrogen bonding, electrostatic forces, complexation, and ion exchange, as discussed below.

#### 6.1.1. Electrostatic attraction

**Fig. 15:**
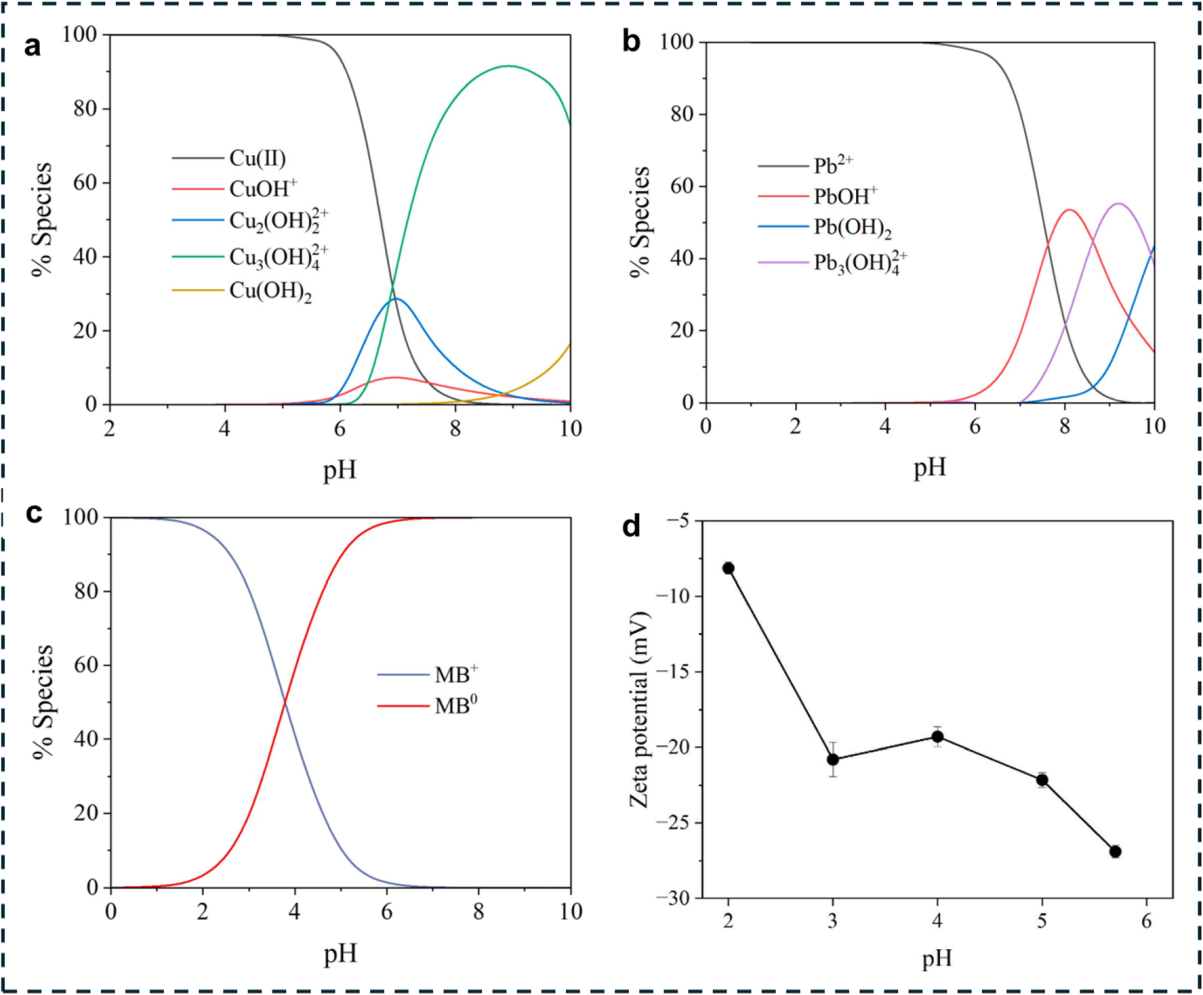
(a) Species distribution diagram of (a) Cu(ii), (b) Pb(ii), (c) MB, and (d) zeta potential of SA/PVA/CG bead.^238^

Electrostatic attraction appears from coulombic interactions between ionic contaminants and the charged surface of adsorbents. Thus, it is strongly dependent on pollutant speciation, ionic strength, and pH-dependent surface charge. Sodium alginate contributes in two ways: (a) crosslinking ions moderate local charge states so that “electrostatics” frequently combines with complexation or ion exchange, and (b) deprotonated COO^−^ generates a diffuse negative potential (rapid initial uptake of cations). For example, the SA/PVA/CG beads exhibited improved removal performance for MB, Pb^2+^, and Cu^2+^ as the pH increased from 2 to 5.^238^ Pb^2+^ and Cu^2+^ were present in ionic form at pH values below 5 (Fig. 15a–c). Similarly, MB dye was cationic due to protonation of amine groups at pH values below 3.8. The aerogel beads were negatively charged as the pH rose from 2 to 5, as depicted in Fig. 15d. Thus, the removal capacities of MB, Pb^2+^, and Cu^2+^ were improved as the pH rose from 2 to 5 because of the electrostatic interaction between anionic aerogel beads and cationic pollutants (MB, Pb^2+^, and Cu^2+^). Electrostatics are modulated differentially and highly based on the type of compositing materials. The compositing of MOFs with SA introduces positively charged metal-node sites.^155^ For instance, SA@ZIF-8 beads exhibit a point of zero charge (pH_pzc_) of 7.9. The results of pH_pzc_ analysis indicated that the surface of SA@ZIF-8 beads was cationic at pH values greater than 7.9 and anionic at pH values less than 7.9. Thus, based on the pH_pzc_ value of beads and cationic MG dye, the removal of MG by cationic beads was due to the electrostatic forces of interaction. Conversely, the compositing of carbonaceous compounds, such as oxidized CNTs and GO, with SA introduces negatively charged oxygenated functional groups; for cationic MB dye, this phenomenon improves adsorption.^239^

#### 6.1.2. Hydrogen bonding

Hydrogen bonding is a relatively weak interaction that arises when electrostatic forces are diminished. The contribution of hydrogen bonding in SA is particularly pronounced for organic compounds and oxyanions, where adsorption cannot be attributed solely to electrostatic attraction. For example, in the removal of PO_4_^3−^ using an SA/Zr hydrogel, the primary mechanism was hydrogen bonding at solution pH values below 4.19.^240^ The hydrogel exhibited a pH_zpc_ value of 4.19. The surface of the SA/Zr hydrogel was positively charged when the pH of the solution was lower than 4.19. The primary PO_4_^3−^ species was H_3_PO_4_ at pH ≤ 2.13, and the electrostatic interaction between H_3_PO_4_ and the adsorbent was negligible. The adsorption of phosphate occurred at this pH due to hydrogen bonding between the OH group of SA/Zr adsorbent and H_3_PO_4_. In pesticides, hydrogen bonding often correlates with π interactions and hydrophobicity. For example, cost-effective β-CD@MSA-BC efficiently adsorbed chlorpyrifos (CPF).^241^ Hydrogen bonding, hydrophobic inclusion (in the β-CD cavity), Fe^3+^ coordination, and π–π interactions were involved in CPF adsorption. The adsorption rate reached 80% after three adsorption–desorption cycles, indicating that hydrogen-bonding-assisted adsorption is practically reusable.

#### 6.1.3. Ion exchange

Ion exchange in sodium alginate happens when compensating ions in the SA gel are replaced by specific ions with a greater driving force or affinity. Thus, it is inherently stoichiometric, highly sensitive to competing ions, and frequently associated with diffusion resistance and alterations in gel architecture (*e.g.*, shrinkage or swelling). For radionuclides and divalent cations, sodium alginates play a significant role in ion replacement. Crosslinker, including Ca^2+^, provides a replaceable active site specifically in guluronate-rich regions. For example, during the adsorption of Cd^2+^, Cu^2+^, and Pb^2+^ by DSA-AAD@Ca^2+^, the ion exchange and chelation interactions occurred between heavy metal ions and nitrogen, oxygen atoms.^242^ Similarly, the proposed adsorption mechanism of radionuclides by the T/G composite involved ion exchange of cesium (Cs^+^) and strontium (Sr^2+^) with Na^+^, as indicated by the pH results. The ion exchange of radionuclide ions with Na^+^ ions into Na_2_Ti_3_O_7_ can be written as

1 Na_2_Ti_3_O_7_ + Sr^2+^ ⇌ SrTi_3_O_7_ + 2Na^+^

2 Na_2_Ti_3_O_7_ + 2Cs ⇌ Cs_2_Ti_3_O_7_ + 2Na^+^

#### 6.1.4. Surface complexation

Surface complexation refers to the generation of complexes by the incorporation of metal centers/surface functional groups with dissolved species of opposite charge. Surface complexation plays a vital role in the binding of SA and its composites with various pollutants, including pesticides, dyes, nuclear metal ions, and heavy metal ions. In the case of the SA biopolymer, the –OH and –COO– serve as Lewis bases that can directly interact with oxyanions and metal cations, and produce complexes that are more specific and stronger than electrostatic attraction and physical adsorption. The carboxyl groups of alginates have been shown to engage directly in metal binding, in which coordination linkages displace loosely associated water or counterions at the adsorbent surface, thereby supporting chemisorption across many systems. According to recent literature, surface complexation is a primary adsorption mechanism, alongside electrostatic interactions and ion exchange, particularly when SA is integrated with fillers, including metal (oxy)hydroxides, carbon-based materials, and clays, which provide additional complexation sites.^36^ Additionally, the selectivity and strength of surface complexation depend on solution conditions (including pH), the type of embedded fillers (*e.g.*, montmorillonite and GO), and the density of functional groups. Surface complexation is more favorable at higher pH, where carboxyl groups are fully deprotonated, thereby improving the adsorption capacity for cationic contaminants (such as Cd^2+^ and Pb^2+^) and anionic species that can bind to multivalent metal sites added to the composite matrix.^37^

### 6.2. Adsorption mechanism for heavy metal ions

The main adsorption mechanisms for radioactive and heavy metal ions, such as U(vi), Hg^2+^, Cu^2+^, Cd^2+^, and Pb^2+^, include electrostatic attraction, ion exchange, and surface complexation. The numerous –OH and –COO^−^ groups in sodium alginate serve as Lewis base ligands that interact with heavy metal ions to create inner-sphere complexes. In calcium-crosslinked SA, the exchange of ions between target metal ions and Ca^2+^ enhances the adsorption process. The primary mechanism is strongly based on the solution pH, as the degree of deprotonation of carboxyl groups and metal speciation affects the availability of binding sites. For example, the adsorption capacity of Cd^2+^ increased as pH rose, peaking at pH 9, whereas the adsorption efficiency of Cr(vi) ions declined sharply with increasing pH.^243^ The zeta potential of SA/PEI-0.25 indicated that the aerogel carries a positive charge at pH below 4.9 and a negative charge at pH above 4.9. Under acidic conditions, the functional groups of the SA/PEI-0.25 aerogel are rapidly protonated, thereby gaining a positive charge. This prevents chelation or coordination with Cd^2+^ ions because of electrostatic repulsion. On the other hand, the numerous positively charged NH_3_^+^ groups on the aerogel surface created a strong electrostatic attraction to anionic Cr(vi) species, such as Cr_2_O_7_^2−^ and HCrO_4_^−^. As pH rose, the deprotonation of the SA/PEI-0.25 aerogel resulted in free groups such as –NH_2_, –OH, and –COOH that bound Cd^2+^ ions *via* chelation. At this point, an electrostatic repulsive force was created between the SA/PEI-0.25 aerogel and Cr(vi) species.

### 6.3. Adsorption mechanism for organic pollutants

The adsorption of organic contaminants involves several concurrent interactions, with their relative significance influenced by aromaticity, hydrophobicity, molecule structure, and surface charge. For cationic dyes such as CV and MB, adsorption primarily occurs *via* electrostatic attraction between negatively charged carboxylate groups of SA and the cationic dye molecules.^244^ On the other hand, anionic dyes require that the SA surface be modified with positively charged polymers such as metal cations, chitosan, or polyethyleneimine, to create positively charged sites. Pesticides and antibiotics show more complex adsorption behavior due to their molecular charge, which varies with pH, based on their p*K*_a_ values. As a result, organic pollutants adsorb *via* surface complexation, hydrophobic interactions, electrostatic attraction, π–π interactions (*e.g.*, with graphene oxide, biochar, or other carbon nanomaterials), and hydrogen bonding.

### 6.4. Adsorption mechanism for anionic pollutants

The adsorption behavior of phosphate notably differs from that of heavy metals, as negatively charged phosphate is repelled electrostatically by pristine SA, which is negatively charged at neutral pH. Phosphate adsorption mainly depends on metal-mediated ligand exchange rather than on electrostatic attraction alone. Using metal oxides such as Al^3+^, Zr^4+^, La^3+^, and Fe^3+^, as well as multivalent metal ions, provides positively charged sites that can form inner-sphere complexes with phosphate *via* ligand exchange. The solution's pH can influence how phosphate ions initially approach through electrostatic attraction, but typically, the dominant factor is the strong formation of metal–phosphate complexes during adsorption.

## 7. Conclusions, challenges, and future direction

SA-based materials are an intriguing category of bioadsorbents that align with the principles of green chemistry and sustainability for the treatment of emerging pollutants. SA offers various benefits, including ease of modification, ease of gelation, biocompatibility, and biodegradability, which have led to rapid development in wastewater treatment over the past decade. This review comprehensively explores the adsorptive properties of SA and SA-based composite materials (*e.g.*, SA-carbon-based material, SA–MXene, SA–clay minerals, SA–MOFs, and SA–other biopolymers composites). SA-based composites are promising adsorbents for removing radioactive metal ions, heavy metal ions, antibiotics, dyes, pesticides, and phosphate ions. Hydrogen bonding, surface complexation, electrostatic interactions, and ion exchange are the primary mechanisms for removing emerging pollutants. Currently, several challenges remain to be thoroughly investigated for progress in this area. Consequently, the following recommendations are suggested for future research:

• A major issue with SA-based materials as adsorbents in real-world water treatment is the notable reduction in their adsorption performance when used with actual wastewater, compared with controlled lab solutions. In real water, high concentrations of interfering or competing ions, including HCO_3_^−^, SO_4_^2−^, Cl^−^, Na^+^, Ca^2+^, and Mg^2+^, often coexist and markedly interfere with contaminant removal. These interfering ions compete with target pollutants for active sites, primarily the –COO^−^ functional groups on the sodium alginate framework, thereby reducing the number of available binding sites. Divalent cations, including Mg^2+^ and Ca^2+^, have a high tendency to bind with –COO^−^, often leading to partial occupation or irreversible ion exchange in the “egg-box” crosslinked network. This process directly lowers the removal performance of SA-based adsorbents for water pollutants. Similarly, natural organic matter (NOM), such as fulvic acid (FA) and humic acid (HA), shows significant adsorption onto SA surfaces through hydrophobic interactions. Future studies should focus on evaluating SA-based adsorbents under real wastewater conditions, where NOM and interfering ions notably decrease adsorption efficiency. Developing dual-crosslinked structures that feature selective functional groups and anti-fouling surfaces is crucial for enhancing their practical use.

• High salinity poses another major hurdle to the effective use of SA-based adsorbents, especially for treating saline wastewater and industrial effluents. Increased ionic strength weakens electrostatic interactions between charged contaminants and SA functional groups by compressing the electrical double layer surrounding the adsorbent surface. As a result, the adsorption capacity and selectivity are often compromised, particularly in systems where electrostatic attraction primarily drives adsorption. Additionally, long-term exposure to saline environments can change the swelling characteristics and structural integrity of sodium alginate hydrogels, potentially further reducing their prolonged adsorption efficiency. Future research should aim to design salt-resistant SA-based adsorbents with stronger contaminant-binding capacity and improved structural stability, thereby ensuring they maintain high adsorption efficiency even in high-ionic-strength environments.

• Leaching of active components is a critical issue that compromises the prolonged stability of sodium alginate-based composite adsorbents. However, various SA-based composite adsorbents composed of functional nanoparticles, such as Fe_2_O_3_ and TiO_2_, and polymeric materials, including PEI, are used to improve mechanical stability, selectivity, and adsorption performance.^245^ However, under alkaline, acidic, or prolonged working conditions, these active components may partially detach from or dissolve in the alginate framework. This leaching process reduces the number of active sites, leading to a gradual decline in adsorption efficiency, and also poses a risk of secondary contamination by releasing nanoparticles or modifier molecules into the treated water. Future studies should focus on designing highly stable composite sodium alginate-based adsorbents by enhancing the immobilization of functional nanoparticles and modifiers through core–shell structures, dual-crosslinking techniques, and covalent grafting to reduce leaching.

• Regenerating sodium alginate adsorbents is crucial for enhancing sustainability and reducing operational costs; nonetheless, it remains a major hurdle to their practical use. Multiple adsorption/desorption experiments often result in a gradual decline in the adsorption rate due to weakening of the alginate framework, loss of active sites, and insufficient desorption of contaminants. Regeneration also produces contaminated eluates that contain chemical regenerants and concentrated pollutants. These eluates need to be further treated before disposal to prevent additional environmental pollution. The supplementary treatment procedures increase process complexity and operational expenses, thereby constraining the widespread use of sodium alginate-based adsorption systems. Future research should aim to develop gentle, eco-friendly regeneration methods that reduce structural damage while preserving high adsorption capacity over extended cycling.

• SA hydrogels possess intrinsic softness and exhibit swelling in aqueous environments, resulting in unreinforced alginate beads easily deforming or collapsing under applied weight. This mechanical stability is further facilitated by multiple adsorption–desorption cycles. Future research should concentrate on strengthening and self-repairing network architectures. For example, integrating dynamic crosslinks can produce a self-repairing gel that improves cyclic performance. Combining with cellulose nanofiber or MXene can enhance stiffness. Similarly, developing double-network hydrogels can significantly boost toughness.

• The binding of alginate is non-specific, based on hydrogen bonding and electrostatic attraction instead of molecular recognition. SA-based adsorbents can uptake similar polar or charged species, but cannot distinguish among pollutants. This limited selectivity is a recognized disadvantage for targeting specific contaminants. To overcome this challenge, researchers should incorporate SA-based adsorbents with molecular recognition elements. For example, introducing specific functional groups can create binding sites for targeting pollutants, such as phosphate binders for pesticides and thiol groups for mercury. Additionally, molecular imprinting in SA can generate cavities that are selective to target pollutants.

## Author contributions

Yawen Li: writing – original draft, writing – review & editing, funding acquisition, supervision. Humaira Parveen: writing – original draft, supervision, formal analysis, Ibrahim Saleem Alatawi: writing – original draft and formal analysis, Sayeed Mukhtar: writing– review & editing, data curation: Sajjad Hussain: writing – original draft, formal analysis: Uzma Faridi: writing – review & editing, and formal analysis. Mona O. Albalawi: writing – original draft and writing – review & editing. Yan Xu: writing – review & editing, data curation, Irfan Ijaz: writing – original draft, writing – review & editing.

## Conflicts of interest

The authors declared there is no conflicts of interest.

## Data Availability

This review summarizes and examines data from previously published studies. No new experimental data have been produced. All relevant data can be found within this article and its references.
